# Astaxanthin Ameliorates Worsened Muscle Dysfunction of MDX Mice Fed with a High-Fat Diet through Reducing Lipotoxicity and Regulating Gut Microbiota

**DOI:** 10.3390/nu16010033

**Published:** 2023-12-21

**Authors:** Ying Chen, Chenjie Ling, Mengting Chen, Liqiang Yu, Jing Yang, Qi Fang

**Affiliations:** 1 Department of Neurology, The First Affiliated Hospital of Soochow University, Suzhou 215006, China; 20214232027@stu.suda.edu.cn (Y.C.); yuliqiang@suda.edu.cn (L.Y.); 2 Department of Clinical Nutrition, Dushu Lake Hospital Affiliated to Soochow University, Suzhou 215124, China; lcj09202022@163.com; 3 Department of Endocrinology and Metabolism, The First Affiliated Hospital of Soochow University, Suzhou 215006, China; 20214232021@stu.suda.edu.cn; 4 Department of Clinical Nutrition, The First Affiliated Hospital of Soochow University, Suzhou 215031, China

**Keywords:** astaxanthin, Duchenne muscular dystrophy, obesity, lipidomics, gut microbiota

## Abstract

Duchenne muscular dystrophy (DMD), a severe X-linked inherited neuromuscular disease, has a high prevalence of obesity. Obesity exacerbates muscle damage and results in adverse clinical outcomes. Preventing obesity helps DMD patients delay disease progression and improve quality of life. Astaxanthin (AX) is a kind of carotenoid which has antioxidant and anti-adipogenesis effects. In this study, male C57BL/10ScSnDmdmdx/J mice were fed with a normal diet, a high-fat diet (HFD), and an HFD containing AX for 16 weeks, respectively. The results showed that AX significantly increased gastrocnemius fiber cross-section area and grip strength, improved treadmill endurance test and mitochondrial morphology, and reduced muscle triglyceride and malonaldehyde levels compared to the HFD. Lipidomic analysis revealed that AX decreased high levels of triglyceride, diglyceride, ceramides, and wax ester induced by HFD. Gut microbiota analysis indicated that AX supplementation failed to alleviate abnormal microbiota diversity, but increased the relative abundances of *Akkermansia*, *Bifidobacterium*, *Butyricicoccus*, and *Staphylococcus*. In conclusion, AX was expected to alleviate disease progression associated with obesity in DMD patients by reducing lipotoxicity and increasing the abundance of beneficial bacteria.

## 1. Introduction

Duchenne muscular dystrophy (DMD) is a severe and progressive neuromuscular disorder characterized by insufficient production of dystrophin due to mutations in the dystrophin gene [1]. This deficiency results in muscle fiber damage, inflammation, impaired regeneration of muscle fibers, and replacement of muscle by fibrotic and adipose tissue, leading to progressive deterioration of muscle mass and function [2,3]. Glucocorticoid treatment is the standard of care in DMD [4] to improve muscle function, delay the loss of ambulation and onset of cardiomyopathy, and prolong survival [5,6,7]. However, this treatment is associated with various complications including weight gain, osteoporosis, and short stature [8,9,10,11].

Lipid metabolism disorders and fatty infiltration are typical pathological changes in DMD muscle [12]. Diseased muscle exhibits excessive accumulation of fibro-adipogenic progenitors (FAPs), which cause fibrosis and fatty replacement [13]. Mitochondrial dysfunction, caused by loss of dystrophin, further contributes to lipid deposition in DMD muscle by impairing glycolipid utilization and enhancing oxidative stress [14,15,16]. Abnormal lipid accumulation in muscle leads to the production of detrimental lipid intermediates [17,18], which in turn exacerbates mitochondrial damage [19,20]. This creates a vicious cycle of lipid metabolism in DMD muscle.

In addition to abnormal lipid metabolism caused by the loss of dystrophin, factors such as long-term hormone use, unhealthy diets, reduced energy expenditure, and decreased mobility due to the disease contribute to obesity in DMD patients [8,21,22,23]. Patients with DMD exhibit a high prevalence of obesity, with approximately 50% of patients developing obesity around the age of 10 years [24,25]. Obesity in DMD patients is associated with worsened physical and lung function, leading to increased risk of fractures and obstructive sleep apnea [25,26,27,28]. The potential mechanisms may be related to further impaired lipid metabolism [29], exacerbating muscle damage and impeding muscle regeneration due to lipotoxicity [30].

Once weight is gained, biologic adaptations and limited physical activity make it extremely difficult to lose weight [8]. Therefore, prevention strategies are crucial to manage weight and improve quality of life for DMD patients. In addition to a healthy diet, the role of phytochemical supplementation in weight management is gaining increasing attention. Astaxanthin (AX), a naturally fat-soluble carotenoid compound, exhibits strong antioxidant activity, and had positive effects on obesity-related diseases by improving glucolipid metabolism, protecting from oxidative damage and regulating the immune system [31]. Muscle redox disturbances and oxidative stress are considered a key pathogenic mechanism and potential therapeutic target in early-onset myopathies [32]. Astaxanthin supplementation has been shown to ameliorate impairment of muscle in mass and function induced by high-fat diets (HFD) [33] and others [34] and is expected to be a strategy for DMD patients in weight management.

Gut microbiota dysbiosis, characterized by reduced microbial diversity and increased maleficent bacteria, has been observed in MDX mice. Microbiota dysbiosis causes gut inflammation and immune dysregulation, which exacerbates DMD muscle damage [35]. An improved strategy targeting gut dysbiosis could help reduce inflammation and rescue muscle strength [36]. Studies have confirmed the probiotic and anti-gut inflammatory properties of AX in mice fed HFD [37,38,39]. Gut microbiota plays a crucial role in connecting food intervention with disease improvement, as it can be directly influenced by diet. Thus, the effect of AX on gut microbiota of DMD patients is worth exploring.

Therefore, the objective of this study was to investigate the effects of AX supplementation on muscle progression in MDX mice fed with a HFD, while exploring the underlying mechanisms related to muscle lipid metabolism and gut microbiota.

## 2. Materials and Methods

### 2.1. Animals and Experiment Design

Eighteen male C57BL/10ScSnDmdmdx/J mice (specific pathogen free) aged 3–5 weeks were purchased from GemPharmatech Biotechnology Co., Ltd. (Nanjing, China). Mice were housed under a regular 12 h light/dark cycle with constant temperature and humidity at the Laboratory Animal Center of Soochow University and were given free access to food and water. All animal experimental procedures were approved by the Soochow University Animal Welfare Committee.

After one-week of adaptation, MDX mice were randomly divided into three groups: a normal diet group (ND, *n* = 6) (15.8% fat), a high-fat diet group (HFD, *n* = 6) (60% fat), and a high-fat diet containing 0.02% AX group (AX, *n* = 6) (60% fat). The components of three diets were listed in Table 1. All the diets were purchased from dyets-cn Co., Ltd. (Wuxi, China). Astaxanthin was purchased from Shanghai Macklin Biochemical Technology Co., Ltd. (Shanghai, China). The experiment lasted for 16 weeks. Body weight and food intake were monitored once a week. At the end of the experiment, grip strength was measured and a treadmill endurance test was performed. Then, mice were sacrificed with 1% pentobarbital sodium (6 μL per gram of body weight) anaesthetization after fasting for 8 h. Blood samples were collected and were separated by centrifugation at 10,000 rpm for 15 min at 4 °C to obtain serum. Muscle (gastrocnemius, tibialis anterior, and quadriceps femori), fat (subcutaneous and epididymal adipose) and fecal samples were collected, and were quickly frozen in liquid nitrogen. All samples were stored at −80 °C for the following analysis.

### 2.2. Grip Strength Measurement

Grip strength was measured using a grip force meter (Jinan YiYan Technology Development Company Limited, Jinan, China). For each trial, mice were placed on the grip plate and pulled backward with even force until loss of grip. Each mouse was given 5 trials with 10 s of rest between trials. The best three of five trials were averaged to generate the final grip strength.

### 2.3. Treadmill Endurance Test

A treadmill endurance test was performed on a mouse treadmill (Shanghai Xinruan Information Technology Co., Ltd., Shanghai, China) set with 5° inclination. Mice were placed on the treadmill belt with an initial speed of 5.4 m/min after 2 h fasting. The belt speed was accelerated at the rate of 1.8 m/min every 12 min until reaching 9 m/min. When a mouse stopped running, it would be sent to the start point and suffered an electric shock (0.1 mA). If a mouse received five or more electric shocks within 2 min, the mouse was considered exhausted, and the test was over. The distance and time on the belt were recorded at exhaustion for each mouse.

### 2.4. Determination of Serum Lipids and Glucose

After anesthesia, blood samples from the caudal vein were collected to immediately determine the fasting blood glucose (FBG) using a Roche blood glucose meter (F. Hoffmann-La Roche Ltd., Basel, Switzerland). Serum samples stored at −80 °C were dissolved on ice. Serum triglyceride (TG), total cholesterol (TC), and low-density lipoprotein (LDL) levels were determined according to the corresponding instructions of commercial assay kits (Nanjing Jiancheng Bioengineering Institute, Nanjing, China).

### 2.5. Determination of Muscle TG and MDA

A total of 10 mg of gastrocnemius (Gas) samples were weighed and added to PBS at 1:10 (*w*:*v*, mg:μL). After fully grinding, the mixtures were centrifuged at 10,000 rpm for 10 min at 4 °C. The supernatants were collected for TG and malonaldehyde (MDA) determination based on a TG assay kit (Nanjing Jiancheng Bioengineering Institute, Nanjing, China) and an MDA assay kit (Elabscience Biotechnology Co., Ltd., Wuhan, China), respectively. The final values were corrected by protein levels determined using a BCA Protein Assay Kit (Beyotime Biotechnology Co., LTD, Shanghai, China).

### 2.6. Hematoxylin and Eosin Staining

Fresh Gas and adipose samples were fixed in 4% paraformaldehyde and fixative for adipose tissue (Wuhan Service biotechnology Co., Ltd., Wuhan, China) for 24 h, respectively. After dehydration and vitrification, samples were embedded in paraffin and cut into 4 μm slices. Then, paraffin slices were subject to hematoxylin and eosin (H&E) staining, and observed under a light microscope (Leica, Wetzlar, Germany). The Image-Pro Plus software (Version 6.0) was used to measure cross-section areas of adipocytes and muscle fibers.

### 2.7. Immunofluorescence Staining

Gastrocnemius tissues were quickly frozen in liquid nitrogen and were embedded in optimum cutting temperature (OCT) compound. After blocking with 5% BSA for 60 min, 6 μm cryosections were incubated firstly with anti-Perilipin-1 (Cell Signaling, Danvers, MA, USA) overnight at 4 °C followed by appropriate fluorescent secondary antibodies for 1 h in the dark at room temperature. Subsequently, coverslips were mounted with mounting medium containing DAPI. Immunofluorescent images were captured using a fluorescent microscope with the EVOS M7000 imaging system (Thermo Fisher Scientific, Waltham, MA, USA) and analyzed by the Image-Pro Plus (Version 6.0).

### 2.8. Transmission Electron Microscopy Analysis

Fresh Gas samples were cut into 1 mm^3^ of cubes and then fixed in a pre-cooled 2.5% glutaraldehyde solution at 4 °C for 24 h followed by 0.1 M PBS containing 1% osmic acid at room temperature for 2 h. After dehydration with gradient acetone and resin penetration, samples were embedded in embed 812 and polymerized at 60 °C for 48 h. Thin sections with 60–80 nm, cut by an ultramicrotome (Leica, Wetzlar, Germany) with a diamond knife (Daitome, Switzerland), were stained with 2% uranyl acetate in ethanol and 2.6% lead citrate. Images were obtained by a transmission electron microscope (HITACHI, Tokyo, Japan).

### 2.9. Muscle Lipidomic Analysis

Lipid extraction and liquid chromatograph mass spectrometry (LC-MS)-based lipid detection was performed by Suzhou PANOMIX Biomedical Tech Co., Ltd. (Suzhou, China). Gastrocnemius samples (100 mg) were homogenized in a 750 μL chloroform-methanol solution (2:1, *v*/*v*, −20 °C). After homogenization, the mixtures were left to stand for 40 min, vortexed, and then centrifuged for 5 min at 12,000 rpm at room temperature. A total of 300 μL of organic layers were collected and dried in vacuum. Finally, the lipid extracts were redissolved in 200 μL of isopropanol and filtered by 0.22 μm membrane for LC-MS analysis.

LC analysis was performed on a Vanquish UPLC System (Thermo Fisher Scientific, USA) using 2 μL of samples. Chromatographic conditions: UPLC: column, CQUITY UPLC^®^ BEH C18 (1.7 μm, 2.1 × 100 mm); solvent system, A2: acetonitrile/water (60:40, *v*:*v*, 0.1% formic acid, 10 mM ammonium formate), B2: isopropanol/acetonitrile (90:10, *v*:*v*, 0.1% formic acid, 10 mM ammonium formate); gradient program, 0~5 min, 70~57% of A2; 5~5.1 min, 57%~50% of A2; 5.1~14 min, 50%~30% of A2; 14~14.1 min, 30% of A2; 14.1~21 min, 30%~1% of A2; 21~24 min, 1% of A2; 24~24.1 min, 1%~70% of A2; 24.1~28 min, 70% of A2; flow rate, 0.25 mL/min; temperature, 50 °C. The qualitative and quantitative analysis of lipid profiling was performed by an ESI mass spectrometer (Thermo Fisher Scientific, USA) in positive ion and negative ion modes. The orbitrap analyzer scanned over a mass-to-charge ratio (*m*/*z*) range of 150–2000 at a mass resolution of 35,000. Raw data from MS were analyzed by LipidSearch software (version 4.2.28). Annotated lipids were identified putatively based on matching precursor ion *m*/*z* values and ion pattern. Peak quantification was corrected by sum peak normalization for comparation among different magnitudes. Principal component analysis (PCA0, orthogonal projections to latent structures discriminant analysis (OPLS-DA), Venn diagrams, and cluster heatmaps were finished on the BioDeep Cloud platform (https://www.biodeep.cn (accessed on 4 December 2023)).

### 2.10. Gut Microbiota Analysis

Fecal samples were sent to Suzhou PANOMIX Biomedical Tech Co., LTD (Jiangsu, China) to explore microbiota alterations using 16S rRNA sequencing. Briefly, the total DNA of bacteria was extracted with DNeasy Power Soil Kit from Mo Bio/QIAGEN company (Omega Bio-Tek, Norcross, GA, USA). A fluorescence spectrophotometer was used to measure the concentration of extracted DNA sample. DNA quality was determined by 1% agarose gel electrophoresis. After PCR amplification of variable region gene, the amplified products were purified with beads and quantified by fluorescence. Then, the sequencing libraries was prepared by a TruSeq Nano DNA LT Library Prep Kit for paired-end sequencing on an Illumina NovaSeq 6000 platform. QIIME2 software (Version 2019.4), based on the DADA2 method, was used on the data to filter, denoise, merge, and remove chimeras. After dereplication, amplicon sequence variants (ASVs) were obtained for species taxonomy annotation, which was carried out by QIIME2 software (Version 2019.4) with Greengenes database (Release 13.8, http://greengenes.secondgenome.com/ (accessed on 4 December 2023)). The Chao1, Pielou’s evenness, Shannon index, Simpson index and Bray–Curtis-based principal coordinate analysis (PCoA) were conducted with QIIME software (Version 2019.4). Linear discriminant analysis effect size (LEfSe, Galaxy version 1.0) was employed to identify significant differential bacteria between groups. R software (Version 4.2.2) was used for plotting.

### 2.11. Statistical Analysis

The data were analyzed with SPSS v19.0 and presented as mean ± SD. Comparisons among groups were statistical analyzed by one-way analysis of variance (ANOVA) based on the LSD test or Tambane’s T2 test. Lipid comparisons between two groups were analyzed by the independent sample t-test. The false discovery rate was calculated using the Benjamini–Hochberg method. The significantly differential lipids were screened out with the standard of variable important in projection (VIP) > 1 and fold change (FC) ≥ 2 or ≤0.5. Comparisons of Chao1, Pielou’s evenness, Shannon, and Simpson among groups were analyzed by Kruskal–Wallis rank sum test with Dunn’s test. *p* < 0.05 was considered statistically significant.

## 3. Results

### 3.1. The Effects of AX on Body Weight, Body Fat, and Glucolipid Metabolism of MDX Mice Fed with HFD

**Figure 1:**
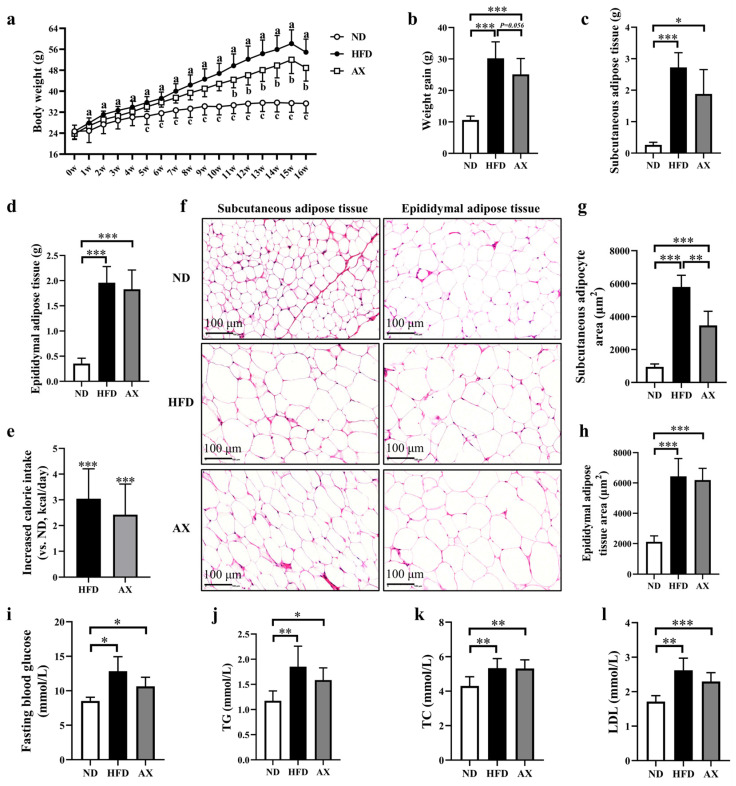
The effects of AX supplementation on body weight, body fat, and glucolipid levels of obese MDX mice. (**a**) Growth curves of the body weight. (**b**) Final weight gain. (**c**) Subcutaneous fat masses. (**d**) Epididymal fat masses. (**e**) Analysis of calorie intake compared with the HFD group. (**f**) H&E staining of adipose tissue. Scale bar: 100 μm. (**g**) Analysis of subcutaneous adipocyte area. (**h**) Analysis of epididymal adipocyte area. (**i**) Fasting blood glucose levels. (**j**) TG levels. (**k**) TC levels. (**l**) LDL levels. HFD vs. ND, ^a^ *p* < 0.05; HFD vs. AX, ^b^ *p* < 0.05; AX vs. ND, ^c^ *p* < 0.05; * *p* < 0.05; ** *p* < 0.01; *** *p* < 0.001.

High-fat diet feeding significantly increased the body weight of MDX mice from week 2, which was effectively alleviated by AX intervention from week 11 (Figure 1a). After the experiment, body weight, subcutaneous, and epididymal fat masses were increased by 184.5%, 570.6%, and 263.2%, respectively, in the HFD group compared with the ND group (Figure 1b–d). Astaxanthin intervention reduced HFD-induced body weight gain and subcutaneous fat mass by 16.8% (*p* = 0.054) and 24.0%, respectively (Figure 1b,c). H&E staining was performed on subcutaneous and epididymal adipose tissues to observe the adipocyte size, and the result was consistent with fat mass (Figure 1f–h). Calorie intake was increased in the HFD group and was decreased after AX intervention (Figure 1e). Astaxanthin had little effect on improving the abnormal increases in FBG, serum TG, TC, and LDL caused by HFD feeding (Figure 1i–l).

### 3.2. The Effects of AX on Muscle Mass, Morphology and Function of MDX Mice Fed with HFD

**Figure 2:**
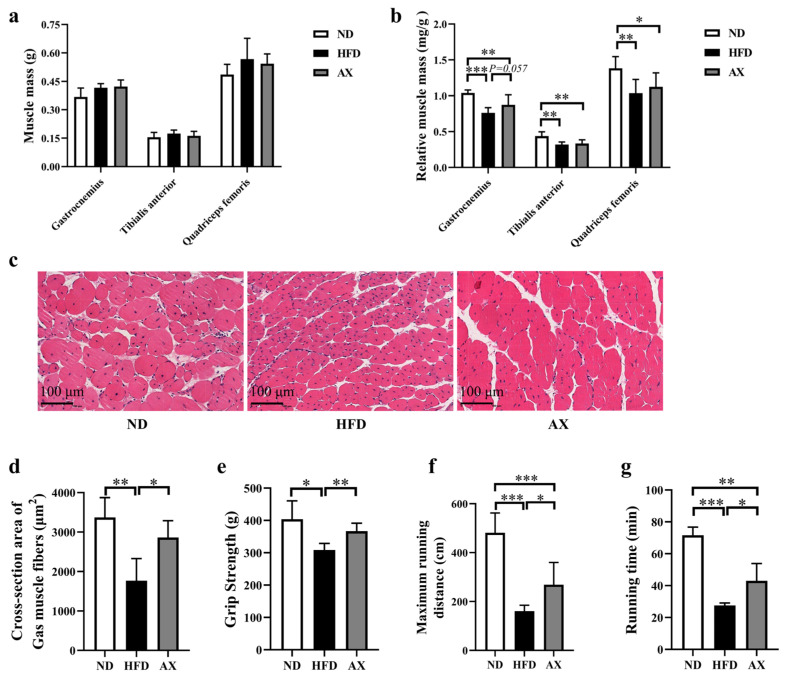
The effects of AX supplementation on muscle mass and function of obese MDX mice. (**a**) Muscle masses of Gas, TA, and Qua. (**b**) Relative muscle masses of Gas, TA, and Qua. (**c**) H&E staining of Gas. Scale bar: 100 μm. (**d**) Analysis of cross-section area of Gas muscle fibers. (**e**) Grip strength at the end of the 16-week intervention. Maximum running distance (**f**) and running time (**g**) of treadmill endurance test after the 16-week intervention. * *p* < 0.05; ** *p* < 0.01; *** *p* < 0.001. Gas, gastrocnemius muscle; TA, tibialis anterior; Qua, quadriceps femoris muscle.

Gastrocnemius, tibialis anterior (TA), and quadriceps femoris (Qua) muscles were weighed, and the results showed no statistical difference among the three groups (Figure 2a). HFD feeding significantly reduced relative masses of the Gas, TA and Qua by 26.7%, 27.3%, and 25.0%, respectively. Astaxanthin supplementation increased Gas relative mass by 14.6% (*p* = 0.057, Figure 2b). Gastrocnemius H&E staining showed that HFD accelerated muscle fiber atrophy, which was improved after AX intervention (Figure 2c). Cross-section area (CSA) of muscle fibers was measured, and the results showed that the CSA of muscle fiber was the smallest in the HFD group, followed by the AX group (Figure 2d). Grip strength and treadmill endurance test were used to evaluate muscle strength and endurance of MDX mice, respectively. Astaxanthin intervention significantly elevated grip strength, maximum running distance, and running time, which were reduced due to HFD feeding (Figure 2e–g).

### 3.3. The Effects of AX on Muscle Lipid Deposition and Mitochondrial Damage of MDX Mice Fed with HFD

**Figure 3:**
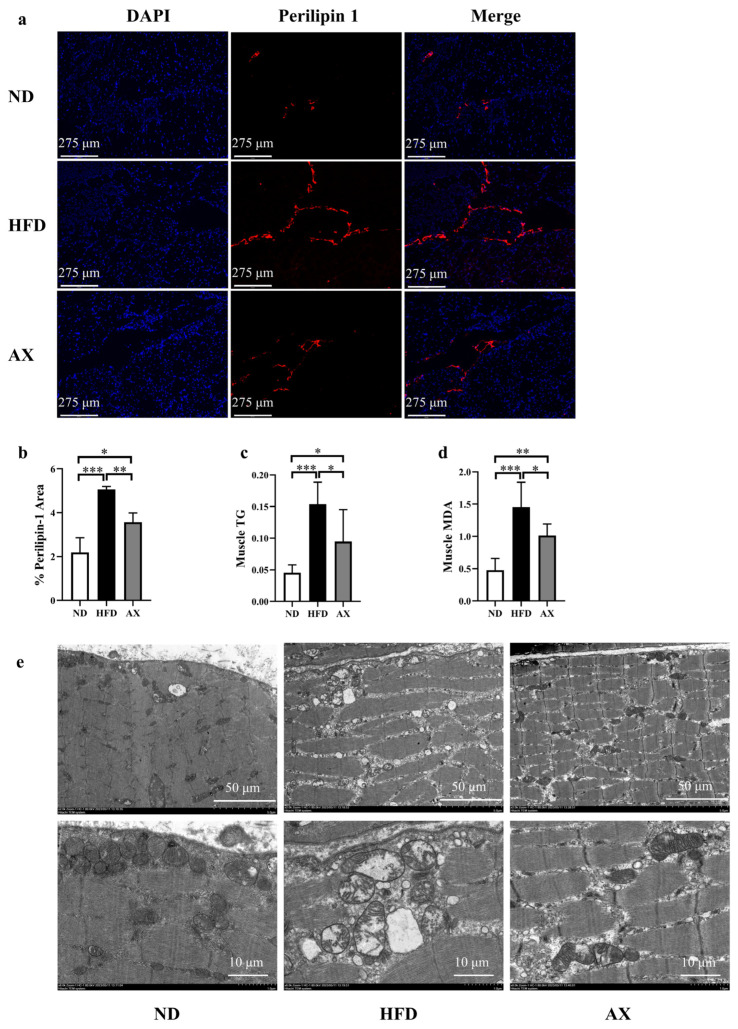
The effects of AX supplementation on muscle fat and mitochondria of obese MDX mice. (**a**) The expression of perilipin 1 in Gas sample under immunofluorescence. Perilipin 1 is indicated by red and nuclei are in blue. Scale bar: 275 μm. (**b**) % Perilipin 1 area; (**c**) Gas TG levels. (**d**) Gas MDA levels. (**e**) Micro photographs of the Gas mitochondria. bar: 1 μm and 5 μm. * *p* < 0.05; ** *p* < 0.01; *** *p* < 0.001. Gas, gastrocnemius muscle.

As shown in Figure 3a–c, HFD feeding significantly increased intermuscular fat and muscle TG by 131.4% and by 238.8%, which were both improved by AX intervention. Mice in the AX group had a 38.8% lower muscle TG than those in the HFD group. Muscle MDA was increased in the HFD group and was decreased in the AX group (Figure 3d). Electron microscopy imaging revealed severe damage to mitochondrial structure in the HFD group, manifested by a loss of cristae and enlarged mitochondria. Astaxanthin intervention effectively alleviated abnormal mitochondrial structure induced by HFD (Figure 3e).

### 3.4. AX Supplementation Improved Muscle Lipid Metabolism by Lipidomic Analysis

**Figure 4:**
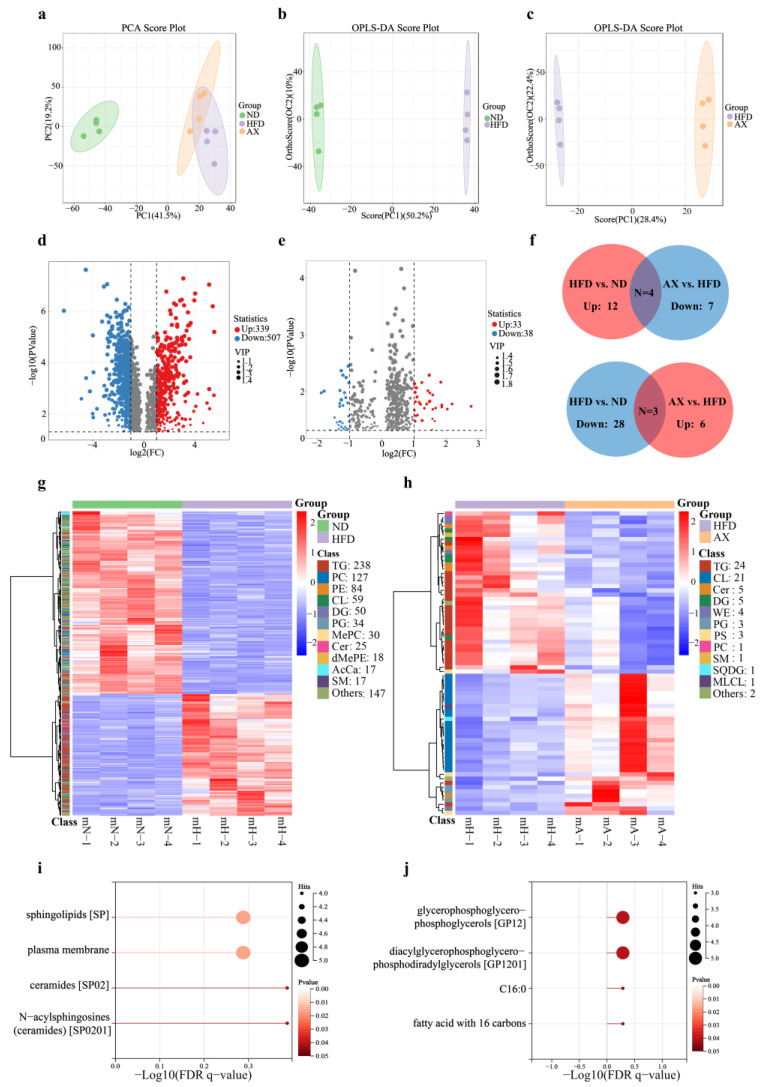
The effects of AX supplementation on lipidomics. Analysis of the PCA plot (**a**); OPLS-DA scores plot (**b**,**c**) and volcano plot (**d**,**e**) analysis of HFD vs. ND and AX vs. HFD, respectively. In volcano plots, significantly differential lipid species were shown as a red (up) or blue (down) dot, while a grey dot represented no significant difference of lipids; Venn plot (**f**) and cluster heatmap (**g**,**h**) analysis of significantly differential lipids in HFD vs. ND and AX vs. HFD; LION lipid functional enrichment analysis of shared significantly differential lipids between HFD vs. ND and AX vs. HFD: (**i**) shared significantly differential lipids up-regulated in HFD vs. ND and down-regulated in AX vs. HFD, and (**j**) shared significantly differential lipids up-regulated in AX vs. HFD and down-regulated in HFD vs. ND. AcCa, Acyl Carnitine; Cer, Ceramides; CL, Cardiolipin; DG, Diglyceride; dMePE, Dimethylphosphatidylethanolamine; MePC, Methylatedphosphatidylcholine; MLCL, Monolysocardiolipin; PC, Phosphatidylcholine; PE, Phosphatidylethanolamine; PG, Phosphatidylglycerol; PS, Phosphatidylserine; SM, Sphingomyelin; SQDG, Sulfoquinovosyldiacylglycerol; TG, Triglyceride; WE, Wax ester.

Unbiased principal component analysis (PCA) was performed to observe the overall lipid alteration in muscle among the three groups. The result showed that the ND group was separated from the HFD and AX groups, while separation between the HFD and AX was little (Figure 4a). To further explore lipid alterations between two groups, OPLS-DA scores plots were obtained and showed that both the ND and the AX groups were separated from the HFD group with model validation parameters of fitness (R^2^X = 0.602 and R^2^Y = 1) and predictability (Q^2^ = 0.976) in HFD versus ND, as well as fitness (R^2^X = 0.638 and R^2^Y = 1) and predictability (Q^2^ = 0.973) in AX versus HFD, respectively (Figure 4b,c). Volcano plot analysis was used to screen out distinct lipid biomarker candidates with the criteria of FC ≥ 2 or ≤0.5 and VIP > 1. 846 (up: 339 and down: 507) and 71 (up: 33 and down: 38) lipids were significantly changed in HFD versus ND and AX versus HFD, respectively (Figure 4d,e, Supplementary Tables S1 and S2).

A total of 3885 lipid species were detected by LC-MS in Gas tissues, including 982 TGs, 460 phosphatidylcholines (PC), 364 phosphatidylethanolamines (PE), 231 cardiolipins (CL), 175 methylphosphatidylethanolamines (MePE), 160 diglycerides (DG), 152 ceramides (Cer), 144 hexosylceramides (Hex1Cer), 120 sphingomyelins (SM), 118 phosphatidylserines (PS), and others. The cluster heatmap revealed that 846 differential lipids in HFD versus ND included 238 TGs, 127PCs, 84PEs, 59CLs, 50DGs, 34PGs, 25Cers, and others, whereas 71 in AX versus HFD covered 24TGs, 21CLs, 5Cers, 5DGs, 4 wax ester (WE), and others (Figure 4g,h). To further investigate whether the lipid biomarkers were related to the biological activity of AX, we identified seven shared significantly differential lipids under the standards of no less than two lipid species in each. Among them, TGs, DGs, Cers, and Wes were upregulated after HFD and downregulated with AX supplementation, while CLs, PGs, and PSs were on the opposite (Figure 4f).

Species-level analysis of differences in lipid composition were performed, and the results were presented in Table 2. There were 40 shared significantly differential lipid species between HFD versus ND and AX versus HFD. A total of 27 lipid species, mostly from TGs, were upregulated in HFD versus ND and downregulated in AX versus HFD, while 11, mostly from CLs and PGs, were on the contrary. Finally, Lion enrichment analysis was performed according to these shared differential lipid species. A total of 27 lipid species were concentrated on four items: sphingolipids (SP), plasma membrane, ceramides (SP02), and N-acylsphingosines (ceramides, SP0201) (Figure 4i). A total of 11 lipid species were concentrated on four items: glycerophosphoglycerophosphoglycerols (GP12), diacylglycerophosphoglycerophosphodiradylglycerols (GP1201), C16;0, and fatty acid with 16 carbons (Figure 4j).

### 3.5. Gut Microbiota Changes in Diversity and Structure Caused by HFD and AX Intervention

**Figure 5:**
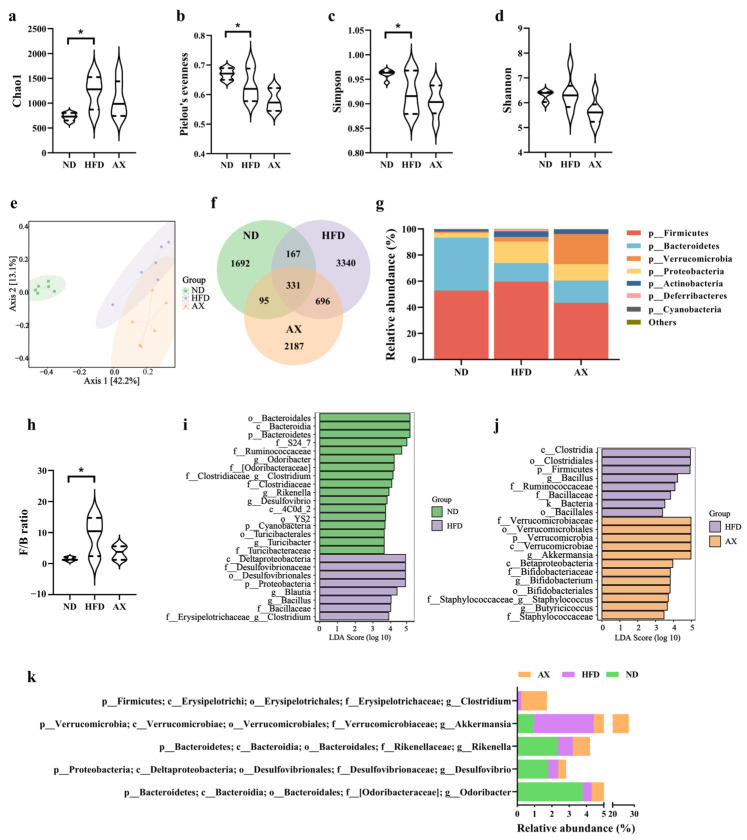
The effects of AX supplementation on gut microbiota of obese MDX mice. Alpha diversity includes (**a**) Chao 1, (**b**) Pielou evenness, (**c**) Simpson index, and (**d**) Shannon index. (**e**) Bray–Curtis based PCoA plot. (**f**) Venn diagram. (**g**) Gut microbiota composition at the phylum level. (**h**) F/B ratio. LDA scores of LEfSE analysis: (**i**) ND vs. HFD and (**j**) HFD vs. AX. (**k**) Differential bacteria at the genus level with average abundance ≥1. * *p* < 0.05.

To investigate the effects of HFD and AX intervention on gut microbiota, 18 colonic content samples from MDX mice in three groups were measured using 16S rRNA sequencing. A total of 1,021,224 effective reads were screened with an average of 56,753 per sample, and 8508 distinct amplicon sequence variants (ASVs) were obtained. HFD feeding increased microbial richness and decreased microbial evenness, as indicated by the significant increase in the Chao1 index and the decrease in Pielou’s evenness index (Figure 5a,b). The Simpson index, an indicator of microbial diversity, was decreased after HFD feeding. Astaxanthin intervention failed to reverse the changes caused by HFD in microbial diversity (Figure 5c,d). There was no significant difference in the Shannon index between the three groups. Principal coordinate analysis (PCoA) was performed to reflect the overall differences of gut microbiota structure among groups via distance. As shown in Figure 5e, the ND group was quite distinct from the HFD and AX groups, whereas the AX group was close to the HFD group. These results indicated that HFD decreased microbial diversity and disordered microbial structure, but AX intervention could not improve the disorder.

The shared and unique ASVs among the three groups were illustrated in the Venn diagram (Figure 5f). Figure 5g showed the gut microbiota abundance at the phylum level. *Bacteroidetes* and *Firmicutes* were most abundant under this level and accounts for 40.47% and 52.84% in the ND group, 14.15% and 59.68% in the HFD group, and 17.04% and 43.42% in the AX group, respectively. The *Firmicutes*-to-*Bacteroidetes* (F/B) ratio was significantly increased in MDX mice fed with HFD, and had a declining trend after AX intervention with no statistical significance (Figure 5h). The differentially dominant taxa in each group were identified by the LEfSe analysis. HFD feeding increased the abundance of *Proteobacteria* at the phylum level, *Desulfovibrionaceae* and *Bacillaceae* at the family level, and *Blautia*, *Bacillus*, and *Clostridium* (from f_*Clostridiaceae*) at the genus level, and decreased the abundance of *Bacteroidetes* and *Cyanobacteria* at the phylum level, *Ruminococcaceae*, *Clostridiaceae*, and *Turicibacteraceae* at the family level, and *Odoribacter*, *Clostridium* (from f_*Erysipelotrichaceae*), *Rikenella*, *Desulfovibrio*, and *Turicibacter* at the genus level (Figure 5i). Compared with the HFD group, AX intervention had higher abundance of *Verrucomicrobia* at the phylum level, *Verrucomicrobiaceae*, *Bifidobacteriaceae*, and *Staphylococcaceae* at the family level, *Akkermansia*, *Bifidobacterium*, *Staphylococcus*, and *Butyricicoccus* at the genus level, and had lower abundance of *Firmicutes* at the phylum level, *Ruminococcaceae* at the family level, and *Bacillaceae* and *Bacillus* at the genus level (Figure 5j). Differential genera with an average abundance ≥1% are shown in Figure 5k, and *Akkermansia* was the only differential bacteria that met the above criteria in the comparison between the AX and HFD groups.

## 4. Discussion

Body mass index is typically higher in children with DMD as compared to normal developing children [24], indicating a susceptibility to obesity in individuals with DMD. Obesity exacerbates disease and increases the risk of obesity-related complications in DMD patients [25,26,27,28]. Therefore, exploring obesity prevention strategies is of great importance for DMD patients. In this study, we observed that AX supplementation was effective in ameliorating further damage of muscle function induced by a HFD in MDX mice. The improvements were attributed to several mechanisms, including improved mitochondria morphology, reduced lipotoxicity, and regulated gut microbiota.

This study revealed that HFD exacerbated the detrimental effects on skeletal muscle mass and function in MDX. The Gas, TA, and quadriceps femoris muscle all decreased in relative mass after HFD feeding. Previous studies have suggested that obesity could worsen skeletal muscle contractile function and muscle regeneration, both of which are pathological processes in DMD [40,41,42,43]. Grip strength and treadmill endurance tests showed a reduction in the HFD group compared to the ND group, indicating further impairment of skeletal muscle contraction function due to HFD. Additionally, the level of adiposity appeared to be a main factor in the magnitude of HFD-induced response [44], indicating that increased fat deposition in skeletal muscle was associated with aggravated damage. In this study, the HFD group exhibited a higher level of skeletal muscle TG in MDX mice. The study by Crabb et al. suggested that HFD was a protective factor in MDX mice, as they observed reduced myofiber necrosis and an increase in running ability without a significant increase in muscle fat deposition after 16 weeks on HFD [45]. The discrepancy in findings could be attributed to different fat content and source. The HFD used by Crabb et al. had a fat content of only 16%, much lower than the 60% used in this study. Additionally, their study primarily used canola oil instead of lard, which was used in this study. Canola oil, rich in unsaturated fatty acids, benefits lipid metabolism. These demonstrated that obesity could exacerbate muscle impairments in MDX mice.

Supplementation of AX significantly improved the decline in muscle function associated with HFD. Nishida et al. found that AX enhanced exercise endurance in HFD-fed mice by promoting mitochondrial biogenesis and alleviating insulin resistance through activation of AMPK pathway [33]. In this study, the cross-section of muscle fibers was increased in the AX group compared to the HFD group, although there were no changes in relative muscle mass. AX has been shown to possess reactive oxygen scavenging ability and anti-inflammatory properties [46], which can reduce oxidative stress and inflammation, known underlying causes of skeletal muscle atrophy and regeneration disorder in both C57BL/6J and MDX mice [35,43,47,48]. We observed that AX reduced the high levels of oxidative stress marker in skeletal muscle induced by HFD. In addition, Kawamura et al. found that AX could elevate protein synthesis and maintain the muscle mass during immobilization-induced atrophy by activating phosphorylation levels of mTOR and p70S6K [49]. In a rat atrophy model, AX was shown to inhibit proteolysis and oxidative stress by decreasing the expression of CuZn-SOD, HSP72, cathepsin L, calpain and ubiquitin in the atrophied muscle [50]. Ren et al. reported that AX enhanced glucose utilization efficiency by targeting the PI3K/Akt/GLUT4 signaling pathway, thereby slowing skeletal muscle atrophy [51]. These suggested that AX could improve oxidative stress and skeletal muscle function in MDX mice.

Increased ectopic fat deposition in muscle can lead to lipotoxicity, charactered by excessive levels of triglyceride and detrimental lipid intermediates such as long-chain acyl CoAs, DGs, and Cers in muscle [17,18]. In this study, HFD led to increased fat deposition in muscle, along with elevated levels of 14 types of Cer and 44 types of DG. Cer and DG were considered key players in lipid-induced insulin resistance and muscle loss [18,52]. Lima et al. found that changes in Cer biosynthesis were common in the muscles of muscle disorder patients, and its accumulation was associated with decline in mitochondrial and protein homeostasis [53]. Inhibiting de novo synthesis of Cers had been shown to improve mitochondrial function and muscle metabolism [53,54]. Astaxanthin alleviated ectopic fat deposition in skeletal muscle and decreased DG (50:0), DG (54:2), Cer (m18:1/18:0), Cer (m18:1/20:0), Cer (m18:1/21:0), and Cer (t42:3), which were increased in the HFD group. Both in vitro and in vitro studies suggested that AX reduced hepatic fat deposition by the improvements in lipogenesis, insulin resistance, inflammation, oxidative stress, apoptosis and endoplasmic reticulum stress. Among these mechanisms, AX more effectively inhibited endoplasmic reticulum stress and lipogenesis compared to other antioxidants [55,56]. These indicated that AX could alleviate skeletal muscle lipotoxicity via reducing fat deposition and detrimental lipid intermediates in MDX mice.

Lipotoxicity causes damage to mitochondria through oxidative stress [19]. Oxidized lipid intermediates reduce mitochondria numbers and function, leading to decreased fatty acid oxidation and increased toxic lipid metabolites [57]. Improvements to mitochondrial pathology were observed to help alleviate destructive processes of DMD muscle [58]. In this study, the HFD-induced lipid toxic environment of muscles resulted in serious damage to mitochondrial morphology, which was improved by AX supplementation. Nishida et al. demonstrated that AX supplementation stimulated mitochondrial biogenesis in insulin resistance muscle via AMPK pathway under HFD condition [33]. The study of Wu et al. showed that AX improved hepatic mitochondrial dysfunction induced by HFD [59]. To summarize, supplementation of AX could improve worsened mitochondrial damage associated with HFD in MDX mice.

Gut microbiota dysbiosis occurs in many diseases, including DMD and obesity [35,36,60]. Compared with the ND group, the HFD group exhibited an increase in Chao1 value, while a decrease in Pielou’s evenness and Simpson values, indicating a further reduction in microbiota diversity in MDX mice with the HFD feeding. Analysis of microbiota composition revealed an increased F/B ratio in the HFD group, consistent with previous studies on HFD-induced microbiota responses [61]. Supplementation of AX failed to improve microbiota diversity and F/B values, but it increased the abundances of beneficial bacteria *Akkermansia*. LefSE analysis identified that *Akkermansia* was the only bacteria with a relative abundance change exceeding 1% in the AX group compared to the HFD group. Numerous studies had reported a growth of *Akkermansia* following AX supplementation [37,38,62]. A decreased abundance of *Akkermansia* was associated with various diseases such as obesity, diabetes and liver steatosis, while *Akkermansia* supplementation exhibited anti-obesity effects and improve metabolic disorders induced by HFD [63,64]. In addition, elevated abundance of *Akkermansia* had been linked to enhanced muscle atrophy associated with aging or cancer [65,66]. Additionally, AX increased the abundances of *Bifidobacterium*, *Butyricicoccus*, and *Staphylococcus* in this study, all of which exhibited average abundances below 1%. *Bifidobacterium* and *Butyricicoccus* had been demonstrated to be associated with improved metabolic function in pathological conditions [67]. Although *Staphylococci* are benign members of the natural flora, many species, such as *Staphylococcus aureus* and *Staphylococcus epidermidis*, have the capacity to be opportunistic pathogens. Given the limited evidence, further studies were needed to elucidate the effects of AX on *Staphylococcus*. Thus, the beneficial effect of AX on gut microbiota was primarily manifested by the enhanced growth of *Akkermansia*, *Bifidobacterium*, and *Butyricicoccus*.

In this study, AX did not lead to statistically significant changes in metabolic parameters, including weight gain, fat mass, FBG, and blood lipids. These could be influenced by low sample sizes and large intra-group differences. On the other hand, these metabolic results indirectly suggested that the improvements in muscle function with AX supplementation were not solely dependent on changes in body fat. Food intake did not significantly differ between the HFD and AX groups in this study, consistent with previous studies that displayed anti-obesity effects of AX in animal models fed a HFD without effecting food and calorie intake [68,69,70]. Mechanisms included inhibiting lipogenesis, promoting lipolysis and enhancing the antioxidant system [69,70]. Ren et al. found that AX assisted in slowing skeletal muscle atrophy in H22 tumor-bearing mice, without effecting food intake [51]. These findings suggested that AX had little effect on appetite, and that metabolic changes resulting from calorie alterations were not the primary mechanisms by which AX worked in MDX mice. Finally. our observation of minimal improvement in muscle mass might contribute to the fact that the loss of muscle mass and function did not occur simultaneously, with muscle mass loss occurring later than function loss [40].

## 5. Conclusions

In summary, our study suggested that AX supplementation enhanced grip strength and exercise endurance, indicating an improvement in muscle function. The possible mechanisms underlying these effects included reduced ectopic fat deposition and lipotoxicity in muscle, improved mitochondrial morphology and regulated gut microbiota.

## Figures and Tables

**Table 1:** The components of three diets (mg/g).

Ingredient	ND	HFD	AX
Casein	200	258	258
L-Cystine	3	4	4
Sucrose	100	89	89
Cornstarch	397.5	-	-
Dyetrose	132	161	161
Lard	-	317	317
Soybean Oil	70	32	32
Cellulose	50	65	65
Mineral Mix	35	58	57.2
Vitamin Mix	10	13	13
Choline Bitartrate	2.5	3	3
Astaxanthin	-	-	0.2
Total	1000	1000	1000
Protein (%)	20.3	20.0	20.0
Carbohydrate (%)	63.9	20.0	20.0
Fat (%)	15.8	60.0	60.0
Energy density (kcal/g)	4.00	5.26	5.26

**Table 2:** Identification of potential shared lipid biomarkers based on the criteria of a FC ≥ 2 or ≤0.5 and VIP ≥ 1.

Lipid Species	Class	Subclass	HFD vs. ND			AX vs. HFD		
			FC	VIP	Trend	FC	VIP	Trend
TG (16:0_11:3_14:1)	GL	TG	9.93	1.33	Up	0.39	1.38	Down
TG (16:0_11:3_16:0)	GL	TG	8.05	1.38	Up	0.49	1.61	Down
TG (16:0_11:4_16:0)	GL	TG	6.17	1.31	Up	0.29	1.57	Down
TG (16:0_11:4_18:1)	GL	TG	2.47	1.10	Up	0.43	1.49	Down
TG (16:1_11:2_14:0)	GL	TG	6.52	1.37	Up	0.41	1.57	Down
TG (18:0e_16:0_18:1)	GL	TG	5.67	1.39	Up	0.41	1.40	Down
TG (18:1_18:1_23:0)	GL	TG	4.06	1.35	Up	0.44	1.34	Down
TG (18:1_18:1_24:0)	GL	TG	4.39	1.34	Up	0.43	1.36	Down
TG (20:1e_16:0_18:1)	GL	TG	3.6	1.34	Up	0.39	1.35	Down
TG (25:0_18:1_18:1)	GL	TG	3.75	1.34	Up	0.45	1.34	Down
TG (26:0_18:1_24:2)	GL	TG	4.55	1.36	Up	0.5	1.36	Down
TG (30:0_18:1_24:2)	GL	TG	5.75	1.36	Up	0.5	1.35	Down
TG (61:2e)	GL	TG	3.57	1.33	Up	0.45	1.37	Down
TG (70:3)	GL	TG	10.89	1.40	Up	0.43	1.40	Down
TG (72:4)	GL	TG	6.61	1.38	Up	0.41	1.37	Down
TG (16:0_16:0_22:6)	GL	TG	0.32	1.31	Down	2.04	1.55	Up
DG (50:0)	GL	DG	2.39	1.27	Up	0.46	1.61	Down
DG (54:2)	GL	DG	3.6	1.34	Up	0.39	1.35	Down
PG (20:5_22:6)	GP	PG	0.23	1.37	Down	2.04	1.43	Up
PG (38:4)	GP	PG	0.46	1.20	Down	2.12	1.44	Up
PG (42:7)	GP	PG	0.43	1.27	Down	2.37	1.54	Up
PC (28:1_18:2)	GP	PC	3.09	1.02	Up	0.38	1.38	Down
CL (18:1_16:0_16:0_18:1)	GP	CL	0.29	1.27	Down	4.52	1.49	Up
CL (18:2_18:1_20:4_16:0)	GP	CL	0.4	1.23	Down	2.99	1.54	Up
CL (18:2_18:1_22:4_18:2)	GP	CL	0.37	1.08	Down	4.09	1.47	Up
CL (18:2_20:4_18:1_18:1)	GP	CL	0.33	1.30	Down	3.55	1.59	Up
CL (69:5)	GP	CL	0.36	1.27	Down	2.5	1.46	Up
SM (t18:0_24:3)	SP	SM	2.92	1.22	Up	0.46	1.52	Down
Cer (d18:2_21:2)	SP	Cer	0.2	1.28	Down	2.94	1.34	Up
Cer (m18:1_18:0)	SP	Cer	8.73	1.38	Up	0.42	1.68	Down
Cer (m18:1_20:0)	SP	Cer	5.86	1.34	Up	0.48	1.50	Down
Cer (m18:1_21:0)	SP	Cer	4.07	1.27	Up	0.42	1.64	Down
Cer (t42:3)	SP	Cer	4.51	1.12	Up	0.27	1.54	Down
CerG2GNAc1 (d42:1 + O)	SL	CerG2GNAc1	0.21	1.25	Down	2.8	1.52	Up
WE (20:1_16:0)	WE	WE	6.15	1.39	Up	0.49	1.65	Down
WE (21:1_16:0)	WE	WE	4.28	1.33	Up	0.4	1.47	Down
WE (22:1_16:0)	WE	WE	5.06	1.33	Up	0.43	1.61	Down
ZyE (33:0)	ZyE	ZyE	2.5	1.16	Up	0.4	1.35	Down
DG (18:2_18:2)	GL	DG	0.46	1.03	Down	0.33	1.33	Down
SQDG (51:12)	GL	SQDG	6.74	1.17	Up	2.01	1.59	Up

Abbreviation: Cer, Ceramides; CerG2GNAc1, Neutral glycosphingolipids; CL, Cardiolipin; DG, Diglyceride; GL, Glycerolipids; GP, Glycerophospholipids; PC, Phosphatidylcholine; PG, Phosphatidylglycerol; SL, Saccharolipids; SM, Sphingomyelin; SP, Sphingolipids; SQDG, Sulfoquinovoyl diacylglycerol; TG, Triglyceride; WE, Wax ester; ZyE, Zymosteryl.

## Data Availability

Data are contained within the article and supplementary materials.

## Supplementary Materials

The following supporting information can be downloaded at: https://www.mdpi.com/article/10.3390/nu16010033/s1, Table S1: Potential lipid biomarkers based on the criteria of a FC ≥ 2 or ≤0.5 and VIP > 1 in HFD vs. ND, Table S2: Potential lipid biomarkers based on the criteria of a FC ≥ 2 or ≤0.5 and VIP > 1 in AX vs. HFD.
