# Supplementary material for: Astaxanthin Ameliorates Worsened Muscle Dysfunction of MDX Mice Fed with a High-Fat Diet through Reducing Lipotoxicity and Regulating Gut Microbiota
Source: Nutrients. 2023 Dec 21;16(1):33. doi: 10.3390/nu16010033 (PMC10780320; doi:10.3390/nu16010033)
Supplement: Supplementary file 1 [file nutrients-16-00033-s001.zip › nutrients-2703601-supplementary.pdf]

Supplementary Table S1: Potential lipid biomarkers based on the criteria of FC ≥ 2 or ≤ 0.5 and VIP > 1 in HFD vs. ND.

**Lipid species**	**Class**	**FC**	**VIP**	**Trend**
BiotinylPE(31:0)	BiotinylPE	2.33	1.27	Up
BisMePA(28:0_18:1)	BisMePA	18.14	1.29	Up
BisMePA(31:0_18:1)	BisMePA	4.12	1.29	Up
BisMePA(40:7e)	BisMePA	2.47	1.38	Up
BisMePA(38:7e)	BisMePA	2.16	1.39	Up
BisMePA(18:0_16:0)	BisMePA	2.09	1.23	Up
BisMePE(16:0_20:4)	BisMePE	2.48	1.37	Up
Cer(m17:1_17:1)	Cer	40.94	1.40	Up
Cer(m18:1_18:0)	Cer	8.73	1.38	Up
Cer(m18:1_20:0)	Cer	5.86	1.34	Up
Cer(d44:0+O)	Cer	4.71	1.04	Up
Cer(t42:3)	Cer	4.51	1.12	Up
Cer(m18:1_21:0)	Cer	4.07	1.27	Up
Cer(m18:0_16:0)	Cer	4.02	1.33	Up
Cer(d18:1_25:0+O)	Cer	3.85	1.01	Up
Cer(m18:1_22:0)	Cer	3.52	1.31	Up
Cer(d43:0)	Cer	3.23	1.01	Up
Cer(m18:1_23:0)	Cer	3.05	1.34	Up
Cer(d44:0)	Cer	2.83	1.01	Up
Cer(m18:1_24:1)	Cer	2.51	1.22	Up
Cer(m18:0_18:0)	Cer	2.18	1.31	Up
CerG3GNAc2(d34:1)	CerG3GNAc2	2.20	1.12	Up
CerPE(d18:1_15:1)	CerPE	2.45	1.03	Up
CL(20:5_22:5_22:6_18:2)	CL	6.63	1.13	Up
CL(79:11)	CL	6.62	1.21	Up
CL(82:16)	CL	5.65	1.09	Up
CL(80:15)	CL	2.96	1.25	Up
CL(84:14)	CL	2.76	1.10	Up
CL(86:12)	CL	2.73	1.32	Up
CL(74:7)	CL	2.64	1.34	Up
CL(18:2_22:5_22:6_18:2)	CL	2.49	1.29	Up
CL(86:14)	CL	2.30	1.34	Up
CL(84:13)	CL	2.28	1.19	Up
CL(83:10)	CL	2.23	1.33	Up
CL(81:8)	CL	2.21	1.37	Up
CL(84:16)	CL	2.14	1.39	Up
CL(85:8)	CL	2.02	1.28	Up
CL(80:11)	CL	2.00	1.32	Up
CL(86:13)	CL	2.00	1.28	Up
DG(34:0)	DG	44.74	1.39	Up
DG(16:0_18:2)	DG	9.46	1.25	Up

DG(22:5_20:4)	DG	8.54	1.34	Up
DG(38:2e)	DG	7.74	1.38	Up
DG(35:2e)	DG	6.26	1.34	Up
DG(36:0e)	DG	6.11	1.40	Up
DG(24:0e)	DG	5.37	1.38	Up
DG(24:2e)	DG	5.15	1.31	Up
DG(26:3e)	DG	4.88	1.38	Up
DG(40:3e)	DG	4.64	1.39	Up
DG(34:0e)	DG	4.33	1.40	Up
DG(36:3e)	DG	4.14	1.40	Up
DG(26:2e)	DG	4.12	1.34	Up
DG(26:1e)	DG	4.03	1.35	Up
DG(28:4e)	DG	4.03	1.35	Up
DG(35:0e)	DG	3.92	1.39	Up
DG(50:1)	DG	3.76	1.37	Up
DG(24:3e)	DG	3.68	1.34	Up
DG(22:0e)	DG	3.66	1.35	Up
DG(54:2)	DG	3.60	1.34	Up
DG(22:6_14:1)	DG	3.46	1.18	Up
DG(40:1e)	DG	3.46	1.35	Up
DG(33:0e)	DG	3.31	1.39	Up
DG(18:1e)	DG	3.07	1.25	Up
DG(22:2e)	DG	3.01	1.32	Up
DG(40:6e)	DG	2.77	1.34	Up
DG(38:1e)	DG	2.70	1.34	Up
DG(40:4e)	DG	2.70	1.34	Up
DG(18:0e)	DG	2.67	1.34	Up
DG(33:3e)	DG	2.60	1.38	Up
DG(16:0_16:1)	DG	2.57	1.23	Up
DG(16:1_18:3)	DG	2.57	1.23	Up
DG(18:0_18:1)	DG	2.50	1.28	Up
DG(16:0_14:0)	DG	2.49	1.11	Up
DG(31:0e)	DG	2.47	1.38	Up
DG(50:0)	DG	2.39	1.27	Up
DG(26:0e)	DG	2.35	1.25	Up
DG(28:3e)	DG	2.35	1.25	Up
DG(20:1e)	DG	2.22	1.25	Up
DG(36:1e)	DG	2.21	1.38	Up
DG(16:0_20:5)	DG	2.18	1.17	Up
DG(38:8e)	DG	2.11	1.17	Up
DG(15:0_18:1)	DG	2.02	1.17	Up
DG(30:3e)	DG	2.02	1.37	Up
DGDG(47:7)	DGDG	3.84	1.00	Up
dMePE(36:4)	dMePE	2.39	1.35	Up

dMePE(18:0e_22:4)	dMePE	2.01	1.24	Up
LBPA(16:0_20:4)	LBPA	2.28	1.14	Up
LdMePE(22:5)	LdMePE	2.33	1.26	Up
LPC(22:5)	LPC	2.16	1.23	Up
LPE(22:5)	LPE	2.30	1.24	Up
MePC(37:3e)	MePC	2.77	1.36	Up
MePC(38:4e)	MePC	2.70	1.11	Up
MePC(38:9)	MePC	2.31	1.29	Up
MePC(29:1e)	MePC	2.25	1.05	Up
MePC(32:3e)	MePC	2.16	1.39	Up
MGDG(34:0)	MGDG	10.10	1.28	Up
MGDG(34:1)	MGDG	3.10	1.25	Up
MGDG(42:8)	MGDG	2.30	1.35	Up
PC(28:1_18:2)	PC	3.09	1.02	Up
PC(32:4e)	PC	2.86	1.18	Up
PC(22:5_13:0)	PC	2.56	1.35	Up
PC(34:9)	PC	2.50	1.27	Up
PC(17:0_18:1)	PC	2.33	1.31	Up
PC(37:4)	PC	2.33	1.31	Up
PC(22:4_22:5)	PC	2.29	1.25	Up
PC(16:0_22:5)	PC	2.25	1.35	Up
PC(18:0_20:2)	PC	2.24	1.30	Up
PC(18:4_22:6)	PC	2.18	1.38	Up
PC(18:3e_18:2)	PC	2.15	1.31	Up
PC(38:8e)	PC	2.15	1.36	Up
PC(38:0e)	PC	2.14	1.26	Up
PC(17:1_20:5)	PC	2.06	1.02	Up
PC(19:5e)	PC	2.05	1.17	Up
PE(20:0p_22:5)	PE	3.61	1.19	Up
PE(47:3)	PE	2.79	1.16	Up
PE(16:0_22:5)	PE	2.66	1.37	Up
PE(20:3_22:3)	PE	2.47	1.26	Up
PE(20:0_20:2)	PE	2.46	1.26	Up
PE(20:2_22:3)	PE	2.41	1.31	Up
PE(36:3e)	PE	2.16	1.39	Up
PE(22:3_22:5)	PE	2.11	1.21	Up
PE(44:10)	PE	2.11	1.20	Up
PE(42:4e)	PE	2.01	1.24	Up
PE(18:0_22:3)	PE	2.00	1.26	Up
PG(22:5_22:5)	PG	2.82	1.24	Up
PG(18:0_22:5)	PG	2.67	1.30	Up
PI(34:1e)	PI	7.93	1.15	Up
PI(16:0_18:1)	PI	3.11	1.22	Up
PS(22:6_22:6)	PS	2.69	1.27	Up

PS(40:8e)	PS	2.62	1.33	Up
PS(38:7)	PS	2.25	1.34	Up
PS(16:0_18:2)	PS	2.21	1.23	Up
PS(42:6e)	PS	2.17	1.20	Up
PS(44:7)	PS	2.05	1.29	Up
SM(t34:0)	SM	3.16	1.39	Up
SM(t18:0_24:3)	SM	2.92	1.22	Up
SM(d18:2_16:0)	SM	2.56	1.20	Up
SM(d36:5)	SM	2.56	1.20	Up
SM(t18:0_23:6)	SM	2.49	1.08	Up
SM(t41:0)	SM	2.35	1.25	Up
SM(t38:0)	SM	2.29	1.13	Up
SQDG(51:12)	SQDG	6.74	1.17	Up
SQDG(48:12)	SQDG	5.11	1.24	Up
SQDG(36:6)	SQDG	4.96	1.32	Up
SQDG(38:5)	SQDG	3.25	1.30	Up
SQDG(36:2)	SQDG	2.28	1.21	Up
StE(38:2)	StE	2.33	1.03	Up
TG(16:0e_9:0_9:0)	TG	44.74	1.39	Up
TG(53:2)	TG	43.97	1.28	Up
TG(19:1_17:1_19:1)	TG	34.86	1.22	Up
TG(15:0_6:0_18:3)	TG	34.15	1.39	Up
TG(16:1_6:0_20:4)	TG	33.77	1.41	Up
TG(57:3)	TG	30.35	1.25	Up
TG(18:1_18:2_20:2)	TG	24.60	1.10	Up
TG(20:2e_14:4_16:0)	TG	16.57	1.23	Up
TG(18:0_6:0_13:0)	TG	15.95	1.40	Up
TG(15:0_6:0_18:1)	TG	15.22	1.40	Up
TG(17:1_17:1_17:1)	TG	14.78	1.19	Up
TG(15:0_6:0_16:0)	TG	12.56	1.40	Up
TG(15:0_18:1_18:2)	TG	12.43	1.08	Up
TG(72:2)	TG	12.30	1.37	Up
TG(18:4_18:1_18:1)	TG	12.25	1.13	Up
TG(16:0_6:0_12:0)	TG	11.70	1.40	Up
TG(20:2e_18:1_20:4)	TG	11.54	1.39	Up
TG(25:1_9:0_9:0)	TG	11.03	1.37	Up
TG(70:3)	TG	10.89	1.40	Up
TG(70:5)	TG	10.82	1.40	Up
TG(28:0_18:0_24:2)	TG	10.04	1.39	Up
TG(16:0_11:3_14:1)	TG	9.93	1.33	Up
TG(19:1_16:0_18:1)	TG	9.85	1.37	Up
TG(12:1e_11:1_11:1)	TG	9.46	1.25	Up
TG(30:0_18:1_24:1)	TG	9.42	1.38	Up
TG(19:1_19:1_19:1)	TG	9.19	1.29	Up

TG(18:0e_6:0_8:0)	TG	9.07	1.32	Up
TG(58:7e)	TG	8.93	1.39	Up
TG(14:0_11:4_14:0)	TG	8.83	1.40	Up
TG(18:2e_16:0_18:1)	TG	8.53	1.18	Up
TG(20:3e_16:0_18:3)	TG	8.53	1.18	Up
TG(18:0_18:0_18:0)	TG	8.46	1.41	Up
TG(51:1)	TG	8.10	1.37	Up
TG(26:1_6:0_14:1)	TG	8.07	1.05	Up
TG(16:0_11:3_16:0)	TG	8.05	1.38	Up
TG(49:8e)	TG	7.91	1.15	Up
TG(9:0_14:0_18:2)	TG	7.91	1.39	Up
TG(70:1)	TG	7.86	1.39	Up
TG(16:0_6:0_16:1)	TG	7.77	1.40	Up
TG(28:0_16:0_24:2)	TG	7.65	1.39	Up
TG(20:0e_16:1_18:1)	TG	7.63	1.37	Up
TG(16:0_18:1_22:0)	TG	7.09	1.40	Up
TG(16:1_16:1_20:1)	TG	6.79	1.35	Up
TG(59:6e)	TG	6.73	1.39	Up
TG(28:1_18:1_23:0)	TG	6.66	1.39	Up
TG(72:4)	TG	6.61	1.38	Up
TG(16:1_11:2_14:0)	TG	6.52	1.37	Up
TG(16:0_6:0_10:0)	TG	6.24	1.30	Up
TG(4:0_15:0_18:1)	TG	6.22	1.35	Up
TG(16:0_11:4_16:0)	TG	6.17	1.31	Up
TG(16:0_18:1_24:0)	TG	6.04	1.40	Up
TG(30:0_18:1_18:1)	TG	6.03	1.39	Up
TG(14:0e_10:1_12:2)	TG	5.91	1.26	Up
TG(26:0_16:0_18:1)	TG	5.90	1.37	Up
TG(30:0_18:1_24:2)	TG	5.75	1.36	Up
TG(16:0_18:1_21:0)	TG	5.70	1.40	Up
TG(18:0e_16:0_18:1)	TG	5.67	1.39	Up
TG(16:0_18:1_23:0)	TG	5.66	1.40	Up
TG(28:0_18:1_24:2)	TG	5.62	1.36	Up
TG(4:0_16:0_16:0)	TG	5.60	1.40	Up
TG(16:0_10:1_16:1)	TG	5.58	1.36	Up
TG(16:1_11:1_12:0)	TG	5.49	1.38	Up
TG(16:0_11:3_18:1)	TG	5.32	1.37	Up
TG(4:0_14:0_16:1)	TG	5.32	1.39	Up
TG(18:0e_16:0_16:0)	TG	5.31	1.23	Up
TG(25:0_16:0_18:1)	TG	5.14	1.41	Up
TG(17:0_6:0_18:2)	TG	4.73	1.38	Up
TG(28:0_16:0_18:1)	TG	4.72	1.37	Up
TG(28:0_20:4_22:1)	TG	4.68	1.34	Up
TG(68:2)	TG	4.64	1.34	Up

TG(30:1_18:1_19:0)	TG	4.58	1.38	Up
TG(16:0_16:0_17:1)	TG	4.57	1.40	Up
TG(26:0_18:1_24:2)	TG	4.55	1.36	Up
TG(68:3)	TG	4.54	1.39	Up
TG(16:0_12:0_14:0)	TG	4.40	1.28	Up
TG(18:1_10:3_18:1)	TG	4.39	1.30	Up
TG(18:1_18:1_24:0)	TG	4.39	1.34	Up
TG(16:0_9:0_18:1)	TG	4.35	1.37	Up
TG(20:0_18:1_18:1)	TG	4.35	1.37	Up
TG(70:6)	TG	4.29	1.40	Up
TG(18:0_18:0_20:1)	TG	4.24	1.38	Up
TG(16:1_12:0_14:0)	TG	4.20	1.34	Up
TG(18:3_14:1_18:2)	TG	4.15	1.35	Up
TG(23:0_10:3_10:3)	TG	4.13	1.16	Up
TG(53:3)	TG	4.10	1.11	Up
TG(18:1_18:1_23:0)	TG	4.06	1.35	Up
TG(16:1_11:1_18:1)	TG	4.01	1.34	Up
TG(30:1_18:1_23:1)	TG	3.93	1.31	Up
TG(16:0_10:1_12:4)	TG	3.89	1.38	Up
TG(9:0_12:0_14:1)	TG	3.88	1.33	Up
TG(57:2e)	TG	3.87	1.36	Up
TG(28:1_16:0_18:1)	TG	3.84	1.37	Up
TG(16:1_14:2_18:1)	TG	3.81	1.38	Up
TG(10:0_12:0_18:1)	TG	3.78	1.34	Up
TG(16:0_9:0_14:0)	TG	3.75	1.35	Up
TG(25:0_18:1_18:1)	TG	3.75	1.34	Up
TG(52:7e)	TG	3.72	1.24	Up
TG(6:0_20:3_22:1)	TG	3.70	1.14	Up
TG(18:1e_16:0_16:0)	TG	3.66	1.37	Up
TG(16:0e_6:0_10:0)	TG	3.64	1.14	Up
TG(16:0_18:1_24:1)	TG	3.62	1.36	Up
TG(20:1e_16:0_18:1)	TG	3.60	1.34	Up
TG(18:1_11:1_12:1)	TG	3.58	1.34	Up
TG(61:2e)	TG	3.57	1.33	Up
TG(16:0_12:0_16:1)	TG	3.55	1.39	Up
TG(16:0_10:3_16:0)	TG	3.50	1.13	Up
TG(28:1_18:1_23:1)	TG	3.40	1.31	Up
TG(53:1e)	TG	3.39	1.40	Up
TG(14:1e_10:1_11:1)	TG	3.32	1.26	Up
TG(70:4)	TG	3.30	1.36	Up
TG(12:1e_6:0_18:1)	TG	3.28	1.35	Up
TG(55:7)	TG	3.27	1.16	Up
TG(18:1_18:1_21:0)	TG	3.26	1.35	Up
TG(30:1_18:1_18:1)	TG	3.26	1.32	Up

TG(29:0_18:1_18:1)	TG	3.25	1.36	Up
TG(44:1)	TG	3.24	1.30	Up
TG(16:0_10:0_14:0)	TG	3.23	1.21	Up
TG(16:0_10:1_10:1)	TG	3.19	1.32	Up
TG(12:1e_6:0_16:0)	TG	3.18	1.36	Up
TG(20:4e_18:1_18:2)	TG	3.18	1.33	Up
TG(4:0_17:1_18:2)	TG	3.11	1.27	Up
TG(16:1_18:1_19:0)	TG	3.10	1.05	Up
TG(16:1_6:0_16:1)	TG	3.09	1.37	Up
TG(19:1_16:0_16:0)	TG	3.08	1.38	Up
TG(30:1_16:0_18:1)	TG	3.07	1.30	Up
TG(16:1_10:1_11:1)	TG	3.06	1.10	Up
TG(20:4e_10:1_10:1)	TG	3.06	1.33	Up
TG(27:0_16:0_18:1)	TG	3.06	1.38	Up
TG(29:1_16:0_18:1)	TG	3.06	1.37	Up
TG(18:2e_18:1_22:0)	TG	3.01	1.25	Up
TG(20:0e_6:0_6:0)	TG	3.01	1.25	Up
TG(15:0_18:1_18:1)	TG	3.00	1.32	Up
TG(20:0e_18:1_18:1)	TG	2.97	1.29	Up
TG(16:0e_16:0_16:0)	TG	2.94	1.13	Up
TG(59:2e)	TG	2.94	1.35	Up
TG(18:1e_16:0_18:1)	TG	2.90	1.21	Up
TG(55:4e)	TG	2.83	1.32	Up
TG(16:1_12:0_16:1)	TG	2.82	1.30	Up
TG(27:0_16:0_16:0)	TG	2.82	1.37	Up
TG(69:2)	TG	2.77	1.35	Up
TG(16:0e_18:1_18:1)	TG	2.75	1.30	Up
TG(4:0_16:0_16:1)	TG	2.73	1.34	Up
TG(16:1_8:0_16:1)	TG	2.68	1.38	Up
TG(30:1_18:1_19:1)	TG	2.60	1.26	Up
TG(20:3e_18:3_18:3)	TG	2.59	1.24	Up
TG(16:0_6:0_13:0)	TG	2.58	1.24	Up
TG(18:1_14:3_17:1)	TG	2.56	1.30	Up
TG(25:1_18:0_18:0)	TG	2.50	1.21	Up
TG(16:1_10:0_16:1)	TG	2.48	1.25	Up
TG(28:1_18:1_18:1)	TG	2.48	1.33	Up
TG(16:0_11:4_18:1)	TG	2.47	1.10	Up
TG(18:3e_16:0_18:1)	TG	2.47	1.28	Up
TG(16:0_16:0_19:0)	TG	2.46	1.32	Up
TG(12:1e_6:0_18:0)	TG	2.44	1.25	Up
TG(20:1e_18:1_18:2)	TG	2.44	1.09	Up
TG(30:0_18:1_22:5)	TG	2.44	1.04	Up
TG(18:2e_18:1_24:0)	TG	2.40	1.03	Up
TG(20:3e_16:0_18:1)	TG	2.34	1.34	Up

TG(11:0_16:1_18:1)	TG	2.33	1.33	Up
TG(27:0_6:0_16:0)	TG	2.30	1.30	Up
TG(16:1_11:2_12:0)	TG	2.28	1.20	Up
TG(29:1_18:1_18:1)	TG	2.26	1.32	Up
TG(28:1_18:1_24:2)	TG	2.23	1.18	Up
TG(16:0_18:1_22:6)	TG	2.22	1.33	Up
TG(16:1_14:3_18:1)	TG	2.20	1.10	Up
TG(19:1_18:2_18:3)	TG	2.20	1.25	Up
TG(26:1_18:1_18:1)	TG	2.19	1.15	Up
TG(12:0e_10:4_18:3)	TG	2.16	1.12	Up
TG(12:0e_6:0_20:4)	TG	2.16	1.31	Up
TG(55:1e)	TG	2.16	1.04	Up
TG(18:1_18:2_20:3)	TG	2.15	1.29	Up
TG(18:3e_18:1_18:3)	TG	2.15	1.25	Up
TG(16:0_12:2_18:2)	TG	2.13	1.28	Up
TG(16:0_14:0_16:1)	TG	2.12	1.33	Up
TG(15:0_14:0_16:1)	TG	2.11	1.35	Up
TG(18:3e_6:0_10:1)	TG	2.11	1.08	Up
TG(16:0e_16:0_22:6)	TG	2.10	1.36	Up
TG(18:0_11:2_12:0)	TG	2.10	1.08	Up
TG(30:1_18:1_24:2)	TG	2.07	1.14	Up
TG(4:0_16:0_20:4)	TG	2.07	1.13	Up
TG(16:1_14:0_16:1)	TG	2.04	1.39	Up
TG(15:0_16:0_18:1)	TG	2.03	1.31	Up
TG(18:0_16:0_16:0)	TG	2.02	1.14	Up
TG(27:1_18:1_18:1)	TG	2.02	1.14	Up
WE(20:1_16:0)	WE	6.15	1.39	Up
WE(22:1_16:0)	WE	5.06	1.33	Up
WE(21:1_16:0)	WE	4.28	1.33	Up
ZyE(32:3)	ZyE	4.48	1.26	Up
ZyE(34:6)	ZyE	4.23	1.39	Up
ZyE(33:6)	ZyE	4.09	1.31	Up
ZyE(33:0)	ZyE	2.50	1.16	Up
ZyE(32:0)	ZyE	2.03	1.23	Up
AcCa(13:0)	AcCa	0.50	1.30	Down
AcCa(8:0)	AcCa	0.50	1.08	Down
AcCa(6:0)	AcCa	0.48	1.27	Down
AcCa(21:1)	AcCa	0.45	1.18	Down
AcCa(22:3)	AcCa	0.44	1.05	Down
AcCa(19:0)	AcCa	0.43	1.10	Down
AcCa(22:2)	AcCa	0.43	1.25	Down
AcCa(22:6)	AcCa	0.43	1.18	Down
AcCa(20:3)	AcCa	0.42	1.22	Down
AcCa(14:3)	AcCa	0.39	1.12	Down

AcCa(15:0)	AcCa	0.37	1.11	Down
AcCa(10:3)	AcCa	0.35	1.33	Down
AcCa(20:5)	AcCa	0.29	1.25	Down
AcCa(17:1)	AcCa	0.26	1.08	Down
AcCa(19:1)	AcCa	0.24	1.14	Down
AcCa(18:3)	AcCa	0.20	1.19	Down
AcCa(18:2)	AcCa	0.16	1.19	Down
BiotinylPE(30:5)	BiotinylPE	0.39	1.28	Down
BiotinylPE(34:9)	BiotinylPE	0.39	1.33	Down
BiotinylPE(28:3)	BiotinylPE	0.28	1.35	Down
BisMePA(18:3e_16:1)	BisMePA	0.50	1.21	Down
BisMePA(40:8e)	BisMePA	0.39	1.39	Down
BisMePA(18:0_22:5)	BisMePA	0.36	1.36	Down
BisMePA(18:3e_15:0)	BisMePA	0.30	1.36	Down
BisMePA(38:1_16:0)	BisMePA	0.30	1.39	Down
BisMePA(30:1_18:3)	BisMePA	0.28	1.34	Down
BisMePA(16:2e_18:3)	BisMePA	0.16	1.34	Down
BisMePA(30:1_18:4)	BisMePA	0.01	1.40	Down
BisMePE(16:0_18:2)	BisMePE	0.49	1.17	Down
BisMePE(18:2_22:6)	BisMePE	0.39	1.38	Down
BisMePE(18:0_18:2)	BisMePE	0.37	1.25	Down
CarE(20:3)	CarE	0.40	1.27	Down
Cer(d18:1_21:0+O)	Cer	0.49	1.20	Down
Cer(d18:2_24:1)	Cer	0.49	1.34	Down
Cer(d18:1_22:2)	Cer	0.44	1.30	Down
Cer(t18:0_18:0)	Cer	0.44	1.24	Down
Cer(d18:2_24:2)	Cer	0.43	1.30	Down
Cer(d18:2_19:0)	Cer	0.42	1.05	Down
Cer(d18:1_20:4)	Cer	0.38	1.37	Down
Cer(d17:1_24:2)	Cer	0.35	1.34	Down
Cer(d18:1_26:2)	Cer	0.35	1.38	Down
Cer(d18:1_19:0)	Cer	0.29	1.27	Down
Cer(d18:2_21:2)	Cer	0.20	1.28	Down
CerG2GNAc1(d34:4)	CerG2GNAc1	0.48	1.17	Down
CerG2GNAc1(d36:3)	CerG2GNAc1	0.46	1.21	Down
CerG2GNAc1(t34:2)	CerG2GNAc1	0.43	1.22	Down
CerG2GNAc1(d38:8)	CerG2GNAc1	0.41	1.28	Down
CerG2GNAc1(d42:1+O)	CerG2GNAc1	0.21	1.25	Down
CerG3GNAc1(d42:3)	CerG3GNAc1	0.38	1.19	Down
CL(20:5_16:0_16:0_18:2)	CL	0.50	1.18	Down
CL(84:12)	CL	0.50	1.33	Down
CL(71:7)	CL	0.48	1.25	Down
CL(78:13)	CL	0.48	1.16	Down
CL(21:0_22:6_16:0_18:1)	CL	0.47	1.33	Down

CL(81:6)	CL	0.47	1.30	Down
CL(82:14)	CL	0.47	1.38	Down
CL(82:15)	CL	0.47	1.36	Down
CL(18:4_18:2_16:1_18:2)	CL	0.46	1.35	Down
CL(81:7)	CL	0.46	1.29	Down
CL(83:11)	CL	0.45	1.34	Down
CL(18:2_16:1_18:2_18:2)	CL	0.44	1.30	Down
CL(72:6)	CL	0.44	1.28	Down
CL(70:1)	CL	0.43	1.19	Down
CL(72:3)	CL	0.43	1.35	Down
CL(77:9)	CL	0.43	1.36	Down
CL(83:13)	CL	0.43	1.35	Down
CL(66:5)	CL	0.42	1.30	Down
CL(77:8)	CL	0.42	1.20	Down
CL(83:5)	CL	0.42	1.26	Down
CL(68:6)	CL	0.41	1.31	Down
CL(72:2)	CL	0.41	1.28	Down
CL(18:2_18:1_20:4_16:0)	CL	0.40	1.23	Down
CL(76:13)	CL	0.40	1.36	Down
CL(81:5)	CL	0.40	1.33	Down
CL(72:4)	CL	0.39	1.32	Down
CL(78:6)	CL	0.39	1.32	Down
CL(18:2_18:1_22:4_18:2)	CL	0.37	1.08	Down
CL(78:7)	CL	0.37	1.32	Down
CL(82:9)	CL	0.37	1.29	Down
CL(69:5)	CL	0.36	1.27	Down
CL(18:2_20:4_18:1_18:1)	CL	0.33	1.30	Down
CL(18:4_20:4_18:2_18:2)	CL	0.32	1.34	Down
CL(78:12)	CL	0.32	1.16	Down
CL(85:13)	CL	0.32	1.35	Down
CL(89:18)	CL	0.32	1.36	Down
CL(77:7)	CL	0.30	1.24	Down
CL(18:1_16:0_16:0_18:1)	CL	0.29	1.27	Down
CL(72:9)	CL	0.29	1.31	Down
CL(74:5)	CL	0.27	1.37	Down
CL(85:12)	CL	0.27	1.28	Down
CL(84:20)	CL	0.07	1.28	Down
CL(82:17)	CL	0.05	1.33	Down
DG(18:2_18:2)	DG	0.46	1.03	Down
DG(34:4e)	DG	0.45	1.28	Down
DG(18:3_18:2)	DG	0.40	1.13	Down
DG(22:5_18:2)	DG	0.36	1.37	Down
DG(18:2_14:1)	DG	0.23	1.33	Down
DG(32:5)	DG	0.15	1.38	Down

DGDG(29:4)	DGDG	0.13	1.35	Down
DLCL(35:4)	DLCL	0.42	1.29	Down
DLCL(37:6)	DLCL	0.42	1.25	Down
DLCL(33:2)	DLCL	0.24	1.37	Down
dMePE(18:2_22:6)	dMePE	0.49	1.27	Down
dMePE(22:5_18:2)	dMePE	0.46	1.37	Down
dMePE(18:0_18:2)	dMePE	0.45	1.30	Down
dMePE(18:1_18:2)	dMePE	0.44	1.26	Down
dMePE(18:1_22:6)	dMePE	0.40	1.39	Down
dMePE(22:5_22:6)	dMePE	0.32	1.38	Down
dMePE(16:1_18:2)	dMePE	0.31	1.25	Down
dMePE(15:0_16:0)	dMePE	0.30	1.28	Down
dMePE(30:2)	dMePE	0.30	1.38	Down
dMePE(36:8)	dMePE	0.29	1.39	Down
dMePE(18:2_18:2)	dMePE	0.27	1.29	Down
dMePE(16:1_16:1)	dMePE	0.26	1.27	Down
dMePE(34:5)	dMePE	0.26	1.39	Down
dMePE(18:3_20:4)	dMePE	0.25	1.37	Down
dMePE(17:0_18:2)	dMePE	0.21	1.28	Down
dMePE(18:3_22:6)	dMePE	0.12	1.41	Down
GM1(m43:6)	GM1	0.45	1.11	Down
Hex1Cer(d15:1_20:4)	Hex1Cer	0.50	1.26	Down
Hex1Cer(t20:1_22:6)	Hex1Cer	0.49	1.29	Down
Hex1Cer(d15:1_22:6)	Hex1Cer	0.47	1.28	Down
Hex1Cer(d18:2_22:5)	Hex1Cer	0.47	1.35	Down
Hex1Cer(t38:2)	Hex1Cer	0.42	1.33	Down
Hex1Cer(t42:5)	Hex1Cer	0.38	1.34	Down
Hex1Cer(d17:0_22:6)	Hex1Cer	0.35	1.37	Down
Hex1Cer(d38:3)	Hex1Cer	0.34	1.37	Down
Hex1Cer(t18:0_20:5)	Hex1Cer	0.33	1.38	Down
Hex1Cer(d35:3)	Hex1Cer	0.30	1.34	Down
Hex1Cer(d16:2_22:5)	Hex1Cer	0.24	1.38	Down
Hex1Cer(t16:1_22:6)	Hex1Cer	0.24	1.22	Down
Hex1Cer(d22:2_20:4)	Hex1Cer	0.14	1.23	Down
Hex2Cer(d18:1_23:0)	Hex2Cer	0.49	1.27	Down
Hex2Cer(d18:1_24:1)	Hex2Cer	0.47	1.33	Down
Hex2Cer(m42:3)	Hex2Cer	0.47	1.37	Down
Hex2Cer(d31:2)	Hex2Cer	0.44	1.22	Down
Hex2Cer(d18:1_24:0)	Hex2Cer	0.36	1.38	Down
Hex3Cer(t34:2)	Hex3Cer	0.48	1.33	Down
Hex3Cer(t38:1)	Hex3Cer	0.38	1.26	Down
LBPA(18:1_18:2)	LBPA	0.50	1.31	Down
LBPA(20:3_20:4)	LBPA	0.50	1.35	Down
LBPA(20:4_20:4)	LBPA	0.32	1.34	Down

LBPA(18:2_18:2)	LBPA	0.25	1.35	Down
LdMePE(17:0)	LdMePE	0.49	1.37	Down
LdMePE(18:3)	LdMePE	0.25	1.39	Down
LdMePE(20:1)	LdMePE	0.25	1.31	Down
LPC(20:1)	LPC	0.45	1.20	Down
LPC(17:1)	LPC	0.44	1.39	Down
LPC(24:1)	LPC	0.44	1.33	Down
LPC(14:0)	LPC	0.43	1.39	Down
LPC(26:1)	LPC	0.43	1.23	Down
LPC(19:0)	LPC	0.38	1.34	Down
LPC(18:3)	LPC	0.31	1.33	Down
LPC(19:1)	LPC	0.31	1.35	Down
LPC(24:2)	LPC	0.27	1.37	Down
LPC(18:3e)	LPC	0.20	1.33	Down
LPC(20:2e)	LPC	0.19	1.31	Down
LPE(18:3e)	LPE	0.22	1.37	Down
LPE(18:3)	LPE	0.18	1.29	Down
MePC(19:2)	MePC	0.50	1.14	Down
MePC(40:4e)	MePC	0.48	1.17	Down
MePC(40:8e)	MePC	0.48	1.12	Down
MePC(34:6e)	MePC	0.47	1.37	Down
MePC(36:3e)	MePC	0.47	1.02	Down
MePC(21:1e)	MePC	0.46	1.31	Down
MePC(34:3)	MePC	0.46	1.35	Down
MePC(40:1)	MePC	0.46	1.06	Down
MePC(32:2e)	MePC	0.44	1.33	Down
MePC(39:2e)	MePC	0.43	1.19	Down
MePC(31:4e)	MePC	0.37	1.39	Down
MePC(32:5)	MePC	0.36	1.33	Down
MePC(36:3)	MePC	0.36	1.33	Down
MePC(34:4)	MePC	0.31	1.40	Down
MePC(40:8)	MePC	0.31	1.38	Down
MePC(43:3)	MePC	0.27	1.30	Down
MePC(30:2)	MePC	0.26	1.40	Down
MePC(33:4)	MePC	0.26	1.33	Down
MePC(30:1)	MePC	0.25	1.32	Down
MePC(36:9)	MePC	0.24	1.38	Down
MePC(38:4)	MePC	0.24	1.16	Down
MePC(29:1)	MePC	0.22	1.39	Down
MePC(38:5)	MePC	0.21	1.32	Down
MePC(31:2e)	MePC	0.18	1.36	Down
MePC(32:6e)	MePC	0.18	1.32	Down
MGDG(38:3e)	MGDG	0.45	1.31	Down
MGDG(46:9)	MGDG	0.41	1.11	Down

MGDG(36:2e)	MGDG	0.40	1.34	Down
MGDG(42:6)	MGDG	0.40	1.38	Down
MGDG(18:1_22:5)	MGDG	0.38	1.37	Down
MGDG(40:7)	MGDG	0.22	1.35	Down
MGDG(44:12)	MGDG	0.16	1.27	Down
MGMG(38:2)	MGMG	0.47	1.32	Down
MGMG(40:2)	MGMG	0.46	1.29	Down
MGMG(38:3)	MGMG	0.45	1.31	Down
MGMG(36:2)	MGMG	0.40	1.35	Down
PC(14:1e_22:6)	PC	0.50	1.36	Down
PC(18:0e_18:2)	PC	0.50	1.16	Down
PC(18:2_23:0)	PC	0.50	1.02	Down
PC(34:4)	PC	0.50	1.34	Down
PC(18:2_20:4)	PC	0.49	1.36	Down
PC(24:0_18:2)	PC	0.49	1.05	Down
PC(32:4)	PC	0.49	1.08	Down
PC(40:0)	PC	0.49	1.00	Down
PC(20:4e_18:2)	PC	0.48	1.36	Down
PC(24:4e)	PC	0.48	1.25	Down
PC(36:2e)	PC	0.48	1.31	Down
PC(44:3e)	PC	0.48	1.16	Down
PC(16:2e_18:1)	PC	0.47	1.08	Down
PC(18:2e_22:6)	PC	0.47	1.31	Down
PC(33:2)	PC	0.47	1.33	Down
PC(16:1e_16:1)	PC	0.46	1.36	Down
PC(19:0_22:4)	PC	0.46	1.35	Down
PC(31:0_11:4)	PC	0.46	1.28	Down
PC(39:5)	PC	0.46	1.39	Down
PC(18:1_22:6)	PC	0.45	1.36	Down
PC(24:1_20:4)	PC	0.45	1.28	Down
PC(28:1)	PC	0.45	1.23	Down
PC(38:2)	PC	0.45	1.40	Down
PC(38:3)	PC	0.45	1.41	Down
PC(17:1_22:6)	PC	0.44	1.23	Down
PC(20:1_18:2)	PC	0.44	1.35	Down
PC(42:5e)	PC	0.44	1.06	Down
PC(10:1e_16:0)	PC	0.43	1.23	Down
PC(17:1_13:0)	PC	0.43	1.30	Down
PC(18:1e_18:2)	PC	0.43	1.33	Down
PC(20:1)	PC	0.43	1.05	Down
PC(20:2_14:1)	PC	0.43	1.28	Down
PC(36:3e)	PC	0.43	1.32	Down
PC(19:0_20:3)	PC	0.42	1.29	Down
PC(19:0_20:4)	PC	0.42	1.38	Down

PC(20:0_18:2)	PC	0.42	1.39	Down
PC(41:4)	PC	0.42	1.32	Down
PC(34:2e)	PC	0.41	1.34	Down
PC(39:3e)	PC	0.41	1.29	Down
PC(39:4)	PC	0.41	1.37	Down
PC(44:12)	PC	0.41	1.26	Down
PC(16:0_19:0)	PC	0.40	1.16	Down
PC(17:0_22:4)	PC	0.40	1.35	Down
PC(19:0_18:1)	PC	0.39	1.36	Down
PC(20:2_22:6)	PC	0.39	1.35	Down
PC(42:8)	PC	0.39	1.31	Down
PC(18:1e_22:5)	PC	0.38	1.33	Down
PC(30:2)	PC	0.38	1.36	Down
PC(46:7)	PC	0.38	1.22	Down
PC(16:0e_18:2)	PC	0.37	1.39	Down
PC(17:1_18:1)	PC	0.37	1.25	Down
PC(21:3e)	PC	0.37	1.12	Down
PC(42:2)	PC	0.37	1.16	Down
PC(44:3)	PC	0.37	1.05	Down
PC(18:1_14:1)	PC	0.36	1.36	Down
PC(18:1_18:2)	PC	0.36	1.38	Down
PC(22:1_22:6)	PC	0.36	1.34	Down
PC(35:2)	PC	0.36	1.40	Down
PC(12:1e_22:6)	PC	0.35	1.39	Down
PC(32:2)	PC	0.35	1.38	Down
PC(38:3e)	PC	0.35	1.36	Down
PC(41:6)	PC	0.35	1.27	Down
PC(19:3e)	PC	0.34	1.30	Down
PC(16:1_18:2)	PC	0.33	1.39	Down
PC(16:1e_20:5)	PC	0.33	1.35	Down
PC(18:4_20:4)	PC	0.32	1.32	Down
PC(19:0_22:6)	PC	0.32	1.30	Down
PC(20:2)	PC	0.32	1.28	Down
PC(35:0)	PC	0.32	1.39	Down
PC(16:1_20:5)	PC	0.31	1.37	Down
PC(18:0_11:1)	PC	0.31	1.31	Down
PC(18:3_20:5)	PC	0.31	1.37	Down
PC(24:2_18:2)	PC	0.31	1.34	Down
PC(44:11)	PC	0.31	1.38	Down
PC(44:5e)	PC	0.31	1.30	Down
PC(46:5)	PC	0.31	1.13	Down
PC(12:1e_18:1)	PC	0.30	1.36	Down
PC(16:0e_20:5)	PC	0.30	1.33	Down
PC(17:0_18:2)	PC	0.30	1.39	Down

PC(19:0_18:2)	PC	0.30	1.40	Down
PC(20:3_22:4)	PC	0.30	1.38	Down
PC(22:5_22:6)	PC	0.30	1.39	Down
PC(34:4e)	PC	0.30	1.34	Down
PC(18:3_13:0)	PC	0.29	1.29	Down
PC(20:1_22:6)	PC	0.29	1.37	Down
PC(22:5_18:2)	PC	0.29	1.40	Down
PC(35:3)	PC	0.29	1.35	Down
PC(18:3_18:2)	PC	0.27	1.36	Down
PC(19:0_22:5)	PC	0.27	1.40	Down
PC(20:5_22:6)	PC	0.27	1.27	Down
PC(31:0e)	PC	0.27	1.35	Down
PC(37:2)	PC	0.26	1.37	Down
PC(17:1_18:2)	PC	0.25	1.40	Down
PC(18:3e_20:4)	PC	0.25	1.34	Down
PC(18:3_20:4)	PC	0.24	1.38	Down
PC(18:2_18:2)	PC	0.23	1.37	Down
PC(33:3)	PC	0.23	1.32	Down
PC(16:1e_20:3)	PC	0.21	1.33	Down
PC(39:2e)	PC	0.21	1.19	Down
PC(33:4)	PC	0.20	1.35	Down
PC(40:9e)	PC	0.19	1.32	Down
PC(43:1e)	PC	0.19	1.32	Down
PC(36:7)	PC	0.18	1.39	Down
PC(18:3_22:5)	PC	0.17	1.39	Down
PC(40:10)	PC	0.15	1.40	Down
PC(18:3_22:6)	PC	0.14	1.40	Down
PC(31:5)	PC	0.14	1.38	Down
PC(40:9)	PC	0.14	1.37	Down
PC(18:3e_22:6)	PC	0.12	1.36	Down
PC(45:4)	PC	0.12	1.32	Down
PC(14:0_18:3)	PC	0.11	1.40	Down
PC(42:1e)	PC	0.06	1.28	Down
PE(44:4)	PE	0.49	1.31	Down
PE(18:1p_16:1)	PE	0.48	1.24	Down
PE(19:0_18:1)	PE	0.47	1.38	Down
PE(20:3_18:2)	PE	0.47	1.37	Down
PE(18:1_18:2)	PE	0.46	1.38	Down
PE(18:1_24:2)	PE	0.46	1.16	Down
PE(18:1e_15:0)	PE	0.46	1.40	Down
PE(20:1_22:6)	PE	0.46	1.37	Down
PE(16:2e_22:6)	PE	0.45	1.31	Down
PE(18:2e_16:1)	PE	0.45	1.28	Down
PE(20:2_22:6)	PE	0.45	1.31	Down

PE(17:0_20:3)	PE	0.44	1.31	Down
PE(19:0_22:4)	PE	0.44	1.35	Down
PE(38:6e)	PE	0.44	1.38	Down
PE(16:1e_20:3)	PE	0.43	1.22	Down
PE(16:1e_20:5)	PE	0.42	1.33	Down
PE(18:3e_20:4)	PE	0.42	1.26	Down
PE(24:0_18:2)	PE	0.41	1.35	Down
PE(16:0_24:1)	PE	0.40	1.35	Down
PE(17:1_16:0)	PE	0.40	1.35	Down
PE(18:3e_22:6)	PE	0.40	1.39	Down
PE(22:4_18:2)	PE	0.40	1.38	Down
PE(22:5_22:6)	PE	0.40	1.37	Down
PE(18:1p_22:5)	PE	0.39	1.39	Down
PE(22:5_22:5)	PE	0.39	1.17	Down
PE(40:5)	PE	0.39	1.38	Down
PE(16:0p_20:5)	PE	0.38	1.21	Down
PE(20:2_22:4)	PE	0.38	1.35	Down
PE(38:0)	PE	0.38	1.18	Down
PE(16:1_18:1)	PE	0.37	1.37	Down
PE(18:0_16:1)	PE	0.37	1.20	Down
PE(18:2e_15:0)	PE	0.37	1.38	Down
PE(20:1_18:1)	PE	0.37	1.40	Down
PE(22:4_22:5)	PE	0.37	1.35	Down
PE(16:1_16:1)	PE	0.34	1.33	Down
PE(16:0p_18:2)	PE	0.33	1.32	Down
PE(16:1_20:5)	PE	0.33	1.38	Down
PE(22:2_22:6)	PE	0.33	1.31	Down
PE(19:0_22:6)	PE	0.32	1.36	Down
PE(20:5_22:6)	PE	0.31	1.37	Down
PE(17:0_18:2)	PE	0.30	1.30	Down
PE(18:1p_15:0)	PE	0.30	1.36	Down
PE(18:2p_22:6)	PE	0.30	1.40	Down
PE(19:0_18:2)	PE	0.30	1.38	Down
PE(20:3p_22:4)	PE	0.30	1.37	Down
PE(40:9e)	PE	0.30	1.40	Down
PE(40:9)	PE	0.29	1.40	Down
PE(19:1_18:1)	PE	0.28	1.25	Down
PE(18:2p_20:4)	PE	0.27	1.38	Down
PE(36:4e)	PE	0.27	1.39	Down
PE(16:0_18:3)	PE	0.26	1.29	Down
PE(18:3_18:2)	PE	0.26	1.39	Down
PE(22:5_18:2)	PE	0.25	1.38	Down
PE(18:1_18:3)	PE	0.24	1.37	Down
PE(20:3p_22:6)	PE	0.23	1.40	Down

PE(42:10e)	PE	0.22	1.40	Down
PE(18:2p_18:2)	PE	0.21	1.35	Down
PE(18:3_20:4)	PE	0.20	1.39	Down
PE(18:2p_22:5)	PE	0.19	1.39	Down
PE(18:3_22:6)	PE	0.18	1.40	Down
PE(18:3_20:3)	PE	0.17	1.39	Down
PE(18:3e_18:2)	PE	0.17	1.40	Down
PE(18:2p_14:0)	PE	0.16	1.34	Down
PE(32:1e)	PE	0.16	1.34	Down
PE(18:3e)	PE	0.15	1.26	Down
PE(40:8e)	PE	0.15	1.39	Down
PE(14:0_18:2)	PE	0.14	1.38	Down
PE(34:5)	PE	0.14	1.38	Down
PE(18:3e_14:0)	PE	0.13	1.37	Down
PE(36:5e)	PE	0.08	1.32	Down
PE(32:3)	PE	0.07	1.35	Down
PE(16:1_18:3)	PE	0.06	1.39	Down
PE(38:8e)	PE	0.03	1.26	Down
PEt(40:6)	PEt	0.46	1.30	Down
PEt(17:0_18:1)	PEt	0.44	1.20	Down
PEt(42:5)	PEt	0.35	1.11	Down
PEt(44:7)	PEt	0.35	1.37	Down
PEt(19:1_18:1)	PEt	0.27	1.19	Down
PEt(42:7)	PEt	0.22	1.20	Down
PG(20:1_22:6)	PG	0.49	1.26	Down
PG(20:2_18:2)	PG	0.49	1.10	Down
PG(18:0_20:2)	PG	0.46	1.38	Down
PG(38:4)	PG	0.46	1.20	Down
PG(16:0_18:2)	PG	0.44	1.22	Down
PG(42:7)	PG	0.43	1.27	Down
PG(20:4_22:6)	PG	0.41	1.30	Down
PG(22:4_20:4)	PG	0.41	1.27	Down
PG(22:4_22:6)	PG	0.40	1.29	Down
PG(22:6_22:6)	PG	0.40	1.28	Down
PG(40:9)	PG	0.40	1.38	Down
PG(15:0_18:1)	PG	0.39	1.36	Down
PG(16:1_18:2)	PG	0.38	1.31	Down
PG(14:0_18:2)	PG	0.37	1.31	Down
PG(16:0_18:3)	PG	0.37	1.27	Down
PG(20:2_20:4)	PG	0.37	1.22	Down
PG(22:4_22:5)	PG	0.32	1.31	Down
PG(45:0)	PG	0.32	1.28	Down
PG(36:3)	PG	0.30	1.39	Down
PG(20:3_18:2)	PG	0.28	1.36	Down

PG(18:2_20:4)	PG	0.27	1.35	Down
PG(37:2)	PG	0.26	1.08	Down
PG(22:5_20:4)	PG	0.24	1.38	Down
PG(34:1)	PG	0.24	1.32	Down
PG(20:5_20:4)	PG	0.23	1.36	Down
PG(20:5_22:6)	PG	0.23	1.37	Down
PG(22:4_18:2)	PG	0.23	1.34	Down
PG(22:5_22:6)	PG	0.23	1.37	Down
PG(22:4_22:4)	PG	0.18	1.25	Down
PG(16:1_22:6)	PG	0.16	1.38	Down
PG(18:2_18:2)	PG	0.16	1.39	Down
PG(18:3_18:2)	PG	0.14	1.39	Down
PI(18:1_22:6)	PI	0.49	1.34	Down
PI(31:0)	PI	0.49	1.29	Down
PI(18:1_22:5)	PI	0.42	1.36	Down
PI(18:2_20:4)	PI	0.41	1.40	Down
PI(33:1)	PI	0.41	1.17	Down
PI(38:3)	PI	0.38	1.35	Down
PI(16:0_20:3)	PI	0.30	1.24	Down
PI(18:1_18:2)	PI	0.24	1.36	Down
PMe(20:1_18:1)	PMe	0.27	1.19	Down
PS(44:9)	PS	0.49	1.13	Down
PS(36:0)	PS	0.41	1.26	Down
PS(40:6e)	PS	0.41	1.27	Down
PS(35:1)	PS	0.36	1.36	Down
PS(39:5)	PS	0.31	1.37	Down
PS(38:7e)	PS	0.26	1.38	Down
PS(33:1)	PS	0.25	1.37	Down
PS(42:11)	PS	0.23	1.41	Down
PS(41:7)	PS	0.20	1.38	Down
PS(35:2)	PS	0.17	1.39	Down
SM(d18:1_24:3)	SM	0.50	1.33	Down
SM(d42:3)	SM	0.47	1.32	Down
SM(d44:6)	SM	0.47	1.36	Down
SM(d37:1)	SM	0.46	1.36	Down
SM(d42:4)	SM	0.46	1.31	Down
SM(d18:2_24:1)	SM	0.45	1.34	Down
SM(d39:4)	SM	0.42	1.27	Down
SM(d44:3)	SM	0.41	1.31	Down
SM(d44:4)	SM	0.28	1.27	Down
SM(d41:3)	SM	0.27	1.36	Down
SQDG(48:5)	SQDG	0.43	1.21	Down
SQDG(38:9)	SQDG	0.29	1.35	Down
SQDG(40:6)	SQDG	0.15	1.08	Down

ST(d44:2)	ST	0.37	1.27	Down
TG(15:0_18:2_18:3)	TG	0.50	1.32	Down
TG(18:1_17:1_18:3)	TG	0.49	1.37	Down
TG(20:0e_18:3_18:4)	TG	0.49	1.35	Down
TG(51:11)	TG	0.48	1.25	Down
TG(53:6)	TG	0.48	1.11	Down
TG(48:10)	TG	0.47	1.33	Down
TG(18:3_18:2_18:2)	TG	0.45	1.29	Down
TG(25:1_18:2_24:2)	TG	0.45	1.12	Down
TG(16:0_17:0_20:5)	TG	0.44	1.25	Down
TG(16:0e_18:1_18:2)	TG	0.44	1.19	Down
TG(16:1e_18:1_22:6)	TG	0.44	1.28	Down
TG(18:0_18:1_22:3)	TG	0.44	1.38	Down
TG(19:0_18:1_18:2)	TG	0.44	1.37	Down
TG(18:2e_18:1_18:1)	TG	0.42	1.30	Down
TG(18:4_16:1_18:3)	TG	0.42	1.25	Down
TG(12:1e_6:0_18:4)	TG	0.40	1.06	Down
TG(20:0e_22:6_22:6)	TG	0.40	1.40	Down
TG(18:3_17:1_18:2)	TG	0.39	1.34	Down
TG(57:11)	TG	0.39	1.24	Down
TG(16:1_18:2_18:3)	TG	0.38	1.31	Down
TG(18:1_18:2_21:1)	TG	0.38	1.32	Down
TG(19:1_10:4_22:6)	TG	0.37	1.38	Down
TG(19:1_18:1_18:2)	TG	0.37	1.37	Down
TG(16:0_18:1_18:3)	TG	0.36	1.29	Down
TG(16:0e_16:1_18:2)	TG	0.36	1.23	Down
TG(18:2_18:2_23:1)	TG	0.36	1.38	Down
TG(18:3_18:3_20:4)	TG	0.36	1.08	Down
TG(72:6)	TG	0.36	1.29	Down
TG(19:1_18:2_18:2)	TG	0.34	1.40	Down
TG(22:5_14:1_22:5)	TG	0.34	1.36	Down
TG(18:0_18:3_20:1)	TG	0.33	1.27	Down
TG(18:1_20:4_20:4)	TG	0.33	1.34	Down
TG(16:0_16:0_22:6)	TG	0.32	1.31	Down
TG(22:5_18:2_18:2)	TG	0.32	1.38	Down
TG(72:8)	TG	0.32	1.26	Down
TG(18:2_18:2_20:4)	TG	0.31	1.38	Down
TG(18:3_18:2_22:6)	TG	0.31	1.19	Down
TG(33:4)	TG	0.31	1.35	Down
TG(6:0_18:1_18:3)	TG	0.31	1.24	Down
TG(18:0_18:1_20:2)	TG	0.30	1.39	Down
TG(57:12)	TG	0.30	1.39	Down
TG(16:0e_18:2_18:2)	TG	0.28	1.31	Down
TG(45:4)	TG	0.27	1.34	Down

TG(59:7e)	TG	0.25	1.33	Down
TG(20:2_20:5_20:5)	TG	0.24	1.30	Down
TG(18:3_17:1_20:5)	TG	0.23	1.38	Down
TG(26:1_11:4_18:2)	TG	0.18	1.39	Down
TG(18:1_18:2_24:2)	TG	0.15	1.39	Down
TG(32:5e)	TG	0.15	1.38	Down
TG(20:0_18:1_20:3)	TG	0.14	1.41	Down
TG(16:1e_16:0_18:2)	TG	0.12	1.29	Down
TG(57:8)	TG	0.06	1.38	Down
TG(18:3_18:2_18:3)	TG	0.04	1.41	Down
TG(18:3_18:3_20:5)	TG	0.04	1.41	Down
WE(3:0_20:3)	WE	0.27	1.31	Down
ZyE(15:0)	ZyE	0.47	1.31	Down

Supplementary Table S2: Potential lipid biomarkers based on the criteria of FC ≥ 2 or ≤ 0.5 and VIP > 1 in AX vs. HFD.

**Lipid species**	**Class**	**FC**	**VIP**	**Trend**
Cer(d18:2_21:2)	Cer	2.94	1.34	Up
CerG2GNAc1(d42:1+O)	CerG2GNAc1	2.80	1.52	Up
CL(18:1_16:0_16:0_18:1)	CL	4.52	1.49	Up
CL(18:2_18:1_22:4_18:2)	CL	4.09	1.47	Up
CL(66:2)	CL	3.63	1.48	Up
CL(18:2_20:4_18:1_18:1)	CL	3.55	1.59	Up
CL(18:3_18:2_18:1_16:0)	CL	3.18	1.46	Up
CL(18:2_18:2_22:6_18:1)	CL	3.11	1.39	Up
CL(18:1_16:0_16:0_18:2)	CL	2.99	1.45	Up
CL(18:2_18:1_20:4_16:0)	CL	2.99	1.54	Up
CL(20:2_18:1_18:2_18:1)	CL	2.77	1.55	Up
CL(18:2_18:1_16:0_18:1)	CL	2.76	1.50	Up
CL(20:2_16:0_18:2_18:1)	CL	2.69	1.56	Up
CL(66:3)	CL	2.63	1.48	Up
CL(69:5)	CL	2.50	1.46	Up
CL(18:1_16:1_16:0_18:2)	CL	2.30	1.48	Up
CL(18:2_18:2_18:2_18:1)	CL	2.24	1.50	Up
CL(73:7)	CL	2.15	1.42	Up
CL(20:2_18:2_18:1_18:2)	CL	2.13	1.53	Up
CL(18:2_18:1_18:1_18:2)	CL	2.10	1.57	Up
CL(18:2_18:1_16:1_18:1)	CL	2.06	1.50	Up
CL(18:2_18:1_18:2_22:5)	CL	2.05	1.38	Up
CL(18:2_16:1_18:2_18:1)	CL	2.02	1.45	Up
MLCL(52:3)	MLCL	2.97	1.39	Up
PG(42:7)	PG	2.37	1.54	Up
PG(38:4)	PG	2.12	1.44	Up
PG(20:5_22:6)	PG	2.04	1.43	Up
PS(18:0_22:5)	PS	3.63	1.46	Up

PS(17:0_22:6)	PS	2.06	1.41	Up
SQDG(51:12)	SQDG	2.01	1.59	Up
TG(16:0_18:1_20:4)	TG	6.82	1.49	Up
TG(16:0_16:0_22:6)	TG	2.04	1.55	Up
WE(13:0_16:3)	WE	2.36	1.58	Up
Cer(m18:1_20:0)	Cer	0.48	1.50	Down
Cer(m18:1_18:0)	Cer	0.42	1.68	Down
Cer(m18:1_21:0)	Cer	0.42	1.64	Down
Cer(t42:3)	Cer	0.27	1.54	Down
DG(16:0_18:3)	DG	0.49	1.40	Down
DG(50:0)	DG	0.46	1.61	Down
DG(54:2)	DG	0.39	1.35	Down
DG(30:5e)	DG	0.36	1.42	Down
DG(18:2_18:2)	DG	0.33	1.33	Down
PC(28:1_18:2)	PC	0.38	1.38	Down
PS(34:0)	PS	0.49	1.44	Down
SM(t18:0_24:3)	SM	0.46	1.52	Down
TG(26:0_18:1_24:2)	TG	0.50	1.36	Down
TG(30:0_18:1_24:2)	TG	0.50	1.35	Down
TG(16:0_11:3_16:0)	TG	0.49	1.61	Down
TG(8:0_18:1_18:2)	TG	0.48	1.34	Down
TG(30:1_18:1_20:2)	TG	0.47	1.34	Down
TG(27:1_18:1_24:2)	TG	0.46	1.34	Down
TG(18:1_11:1_14:2)	TG	0.45	1.55	Down
TG(25:0_18:1_18:1)	TG	0.45	1.34	Down
TG(61:2e)	TG	0.45	1.37	Down
TG(18:1_18:1_23:0)	TG	0.44	1.34	Down
TG(16:0_11:4_18:1)	TG	0.43	1.49	Down
TG(18:1_18:1_24:0)	TG	0.43	1.36	Down
TG(70:3)	TG	0.43	1.40	Down
TG(16:1_11:2_14:0)	TG	0.41	1.57	Down
TG(18:0e_16:0_18:1)	TG	0.41	1.40	Down
TG(72:4)	TG	0.41	1.37	Down
TG(28:1_18:2_24:2)	TG	0.40	1.40	Down
TG(37:2)	TG	0.40	1.50	Down
TG(16:0_11:3_14:1)	TG	0.39	1.38	Down
TG(20:1e_16:0_18:1)	TG	0.39	1.35	Down
TG(28:1_18:1_18:2)	TG	0.37	1.33	Down
TG(16:0_11:4_16:0)	TG	0.29	1.57	Down
WE(20:1_16:0)	WE	0.49	1.65	Down
WE(22:1_16:0)	WE	0.43	1.61	Down
WE(21:1_16:0)	WE	0.40	1.47	Down
ZyE(33:0)	ZyE	0.40	1.35	Down
